# Identification of Iridoid Glucoside Transporters in *Catharanthus roseus*

**DOI:** 10.1093/pcp/pcx097

**Published:** 2017-07-19

**Authors:** Bo Larsen, Victoria L. Fuller, Jacob Pollier, Alex Van Moerkercke, Fabian Schweizer, Richard Payne, Maite Colinas, Sarah E. O’Connor, Alain Goossens, Barbara A. Halkier

**Affiliations:** 1 DynaMo Center, Department of Plant and Environmental Sciences, Faculty of Science, University of Copenhagen, Thorvaldsensvej 40, 1871 Frederiksberg C, Denmark; 2 Department of Plant Systems Biology, VIB, B-9052 Gent, Belgium; 3 Department of Plant Biotechnology and Bioinformatics, Ghent University, B-9052 Gent, Belgium; 4 Department of Biological Chemistry, John Innes Centre, Norwich NR4 7UH, UK

**Keywords:** *Catharanthus roseus*, Iridoid glucosides, Mobile intermediates, Monoterpenoid indole alkaloids, NPF transporters, Pathway orchestration

## Abstract

Monoterpenoid indole alkaloids (MIAs) are plant defense compounds and high-value pharmaceuticals. Biosynthesis of the universal MIA precursor, secologanin, is organized between internal phloem-associated parenchyma (IPAP) and epidermis cells. Transporters for intercellular transport of proposed mobile pathway intermediates have remained elusive. Screening of an *Arabidopsis thaliana* transporter library expressed in *Xenopus* oocytes identified AtNPF2.9 as a putative iridoid glucoside importer. Eight orthologs were identified in *Catharanthus roseus*, of which three, CrNPF2.4, CrNPF2.5 and CrNPF2.6, were capable of transporting the iridoid glucosides 7-deoxyloganic acid, loganic acid, loganin and secologanin into oocytes. Based on enzyme expression data and transporter specificity, we propose that several enzymes of the biosynthetic pathway are present in both IPAP and epidermis cells, and that the three transporters are responsible for transporting not only loganic acid, as previously proposed, but multiple intermediates. Identification of the iridoid glucoside-transporting CrNPFs is an important step toward understanding the complex orchestration of the seco-iridioid pathway.

## Introduction

Plants are brilliant organic chemists and produce a plethora of specialized metabolites such as flavonoids, phenylpropanoids, terpenoids and alkaloids with a myriad of biological properties. Within the terpenoids, the monoterpenoid indole alkaloids (MIAs) constitute a group of chemically diverse specialized metabolites with pharmacological properties and activity against insect pests. The biosynthesis of MIAs is highly complex, with numerous enzymes and a sophisticated spatial organization (Courdavault et al*.* 2014, De Luca et al*.* 2014, Dugé de Bernonville et al*.* 2015).

Madagascar periwinkle (*Catharanthus roseus*) is the most widely utilized plant for studying the orchestration of the MIA pathway. The MIA pathway is localized in at least four different cell types and at least as many subcellular compartments (St-Pierre et al*.* 1999, Mahroug et al*.* 2007, Verma et al*.* 2012, Courdavault et al*.* 2014). Initially, geraniol is produced inside the plastids of internal phloem-associated parenchyma (IPAP) cells, before being exported to the cytosol, where it is converted into loganic acid (Murata et al*.* 2008, Courdavault et al*.* 2014, Miettinen et al*.* 2014). Secologanin is synthesized from loganic acid in the cytosol of the epidermis cells (Miettinen et al*.* 2014). Secologanin and tryptamine are coupled in the vacuole of the leaf epidermis cells to form strictosidine (Supplementary Fig. S1) (Guirimand et al*.* 2011, Courdavault et al*.* 2014, Miettinen et al*.* 2014). The later branches of the MIA pathway are localized to epidermis cells, laticifers or idioblast cells (Courdavault et al*.* 2014).

Recent mining of large-scale transcriptomic data from various tissues, including jasmonate-inducible and epidermis-specific tissues (Murata et al*.* 2008, GÓngora-Castillo et al*.* 2012, Van Moerkercke et al*.* 2013), has resulted in the identification of all genes in the seco-iridoid and strictosidine pathway from geraniol pyrophosphate to strictosidine (Geu-Flores et al*.* 2012, Courdavault et al*.* 2014, Miettinen et al*.* 2014). As a major subsequent breakthrough, heterologous production of secologanin was achieved by reconstitution of the biosynthetic pathway by transient expression of the enzymes in *Nicotiana benthamiana* (Miettinen et al*.* 2014), and by engineering the strictosidine pathway into the yeast *Saccharomyces cerevisiae* (Brown et al*.* 2015).

An important step towards understanding the orchestration of the seco-iridoid part of the MIA pathway in *C. roseus* is to determine the identity of the pathway intermediate that is transported between the IPAP and the epidermis cells. The 7-deoxyloganic acid hydroxylase (7DLH), which produces loganic acid, is localized in the IPAP cells, as evidenced by in situ hybridization and proteomics (Miettinen et al*.* 2014). The next enzyme in the pathway, loganic acid methyltransferase (LAMT), which produces loganin, was identified in the transcriptome isolated from epidermis tissue (Murata et al*.* 2008), and was confirmed by in situ hybridization to be localized in the epidermis (Guirimand et al*.* 2011), where secologanin synthase (SLS) is also located (Irmler et al*.* 2000). Hence, loganic acid was proposed to be the mobile intermediate (Miettinen et al*.* 2014).

Despite substantial progress in the identification of MIA biosynthetic genes in the last few years, knowledge about the transporters responsible for shuttling pathway intermediates and end-products between cells and organelles is only starting to form. Previously, an ABC transporter, CrTPT2, responsible for exporting MIAs from the epidermis to the cuticle, was identified and characterized (Yu and De Luca 2013). Recently, a nitrate/peptide family (NPF) transporter from *C. roseus*, CrNPF2.9, was identified and characterized as an exporter of strictosidine from the vacuole to the cytosol (Payne et al*.* 2017). Additionally, biochemical characterization of transport of MIAs into the vacuolar storage compartment showed that this process was mediated by a proton-driven antiporter, probably belonging to the multi-drug and toxic compound extrusion (MATE) family (Carqueijeiro et al*.* 2013).

It is inherently difficult to identify transporters of specialized metabolites (Nour-Eldin and Halkier 2013, Larsen et al*.* 2017). To date, the approaches used include substrate-induced expression analysis and co-expression analysis with biosynthetic genes and regulatory loci (Shitan et al*.* 2003, Kidd et al*.* 2006, Morita et al*.* 2009, Shoji et al*.* 2009, Hildreth et al*.* 2011, Shitan et al. 2013). Recently, two *Arabidopsis thaliana* (hereafter Arabidopsis) transporters, AtNPF2.10/GTR1 and AtNPF2.11/GTR2 of the NPF family, were identified by screening a library of Arabidopsis transporters expressed in *Xenopus laevis* oocytes for glucosinolate uptake activity (Nour-Eldin et al*.* 2006, Nour-Eldin et al*.* 2012). This approach does not require a priori knowledge about the nature of the transporter and therefore has very broad application possibilities. Interestingly, the glucosinolate transporters belong to the NPF family, a family proposed to encompass transporters of specialized metabolites (Nour-Eldin and Halkier 2013). Similarly, within the NUP/PUP family, an increasing number of specialized metabolite transporters have been identified (Hildreth et al*.* 2011, Zürchner et al*.* 2016).

Using a functional genomics approach based on screening of an Arabidopsis transporter cDNA library expressed in *Xenopus* oocytes, we first identified Arabidopsis transporters capable of importing the iridoid glucoside, loganin. On the basis of phylogenetic relationships, eight orthologous *C. roseus* transporters, belonging to the NPF family, were identified, three of which were capable of transporting multiple iridoid glucosides; 7-deoxologanic acid, loganic acid, loganin and secologanin, in vitro. One transporter displayed high affinity towards loganin, and two transporters showed medium affinity. We propose that the biosynthetic machinery overlaps between cell types and that multiple intermediates of the seco-iridoid pathway are subjected to transport by these three identified CrNPF transporters.

## Results

### Identification of *C. roseus* iridoid glucoside transporters

**Fig. 1:**
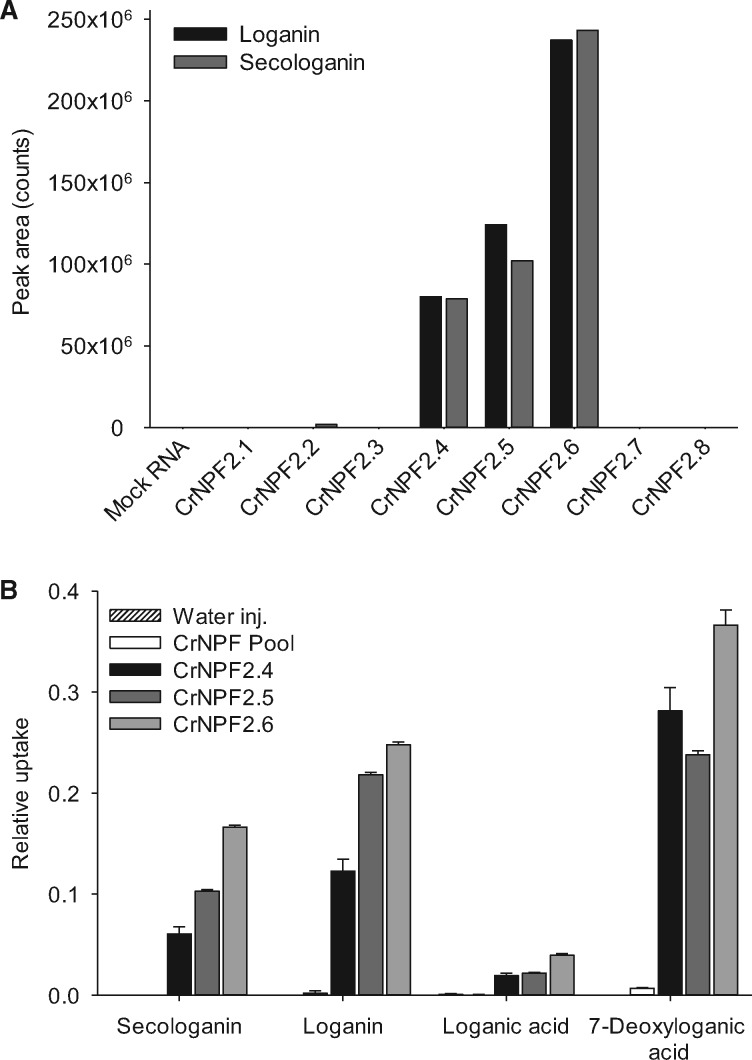
Identification of *C. roseus* transporters with iridoid glucoside uptake activity and determination of substrate specificities in *Xenopus* oocytes. (A) Eight AtNPF2.9 orthologs from *C. roseus* were screened for loganin and secologanin uptake in *Xenopus* oocytes. Transporter-expressing oocytes were exposed to 250 µM substrate for 1 h at pH 5.0. Oocyte extracts were analyzed by LC-MS [*n* = 2 (2 X 5 oocytes)]. (B) The uptake specificities of CrNPF2.4, CrNPF2.5 and CrNPF2.6 were determined using two substrate mixes; a 1 : 1 mix of loganin and secologanin and a 1 : 1 mix of loganic acid and 7-deoxyloganic acid. Oocyte extracts were analyzed for iridoid glucosides by LC-MS, and peak areas of extracted ion chromatograms are depicted directly in (A) and after normalization to compound concentrations in the assay media (media concentration set to 1) in (B). Uptake was measured with substrate concentrations of 250 µM for 30 min at pH 5.0 [error bars are the SE; *n* = 3 (3 X 3 oocytes)]. The CrNPF2 pool comprises the five putative transporters CrNPF2.1, CrNPF2.2, CrNPF2.3, CrNPF2.7 and CrNPF2.8 which tested negative in the initial screen for loganin and secologanin in (A).

To identify iridoid glucoside importers, we first screened 290 Arabidopsis transporter cDNAs, expressed in *Xenopus* oocytes, for uptake activity of the readily obtainable, commercially available iridoid glucoside, loganin. Two transporters were identified: the indole-specific glucosinolate transporter AtNPF2.9 (At1g18880) (JØrgensen et al*.* 2017) and the putative nucleobase ascorbate transporter AtNAT4 (At1g49960). AtNPF2.9 is a member of the NRT1/PTR family (NPF) and a close homolog of the broad-specific glucosinolate transporters AtNPF2.10 and AtNPF2.11 (Nour-Eldin et al*.* 2012, Léran et al*.* 2014). As the NPF family is proposed to encompass long-sought specialized metabolite transporters (Nour-Eldin and Halkier 2013) and is known to include glucoside transporters, we searched for orthologs of the AtNPF2.9 transporter in *C. roseus.* Within the transcriptome sequence of *C. roseus* (Van Moerkercke et al*.* 2013), 40 NPF members were identified*.* Phylogenetic analysis identified a subclade of eight orthologous genes closely related to AtNPF2.9 (Supplementary Fig. S2). All eight transporters were expressed in *Xenopus* oocytes and screened for import of loganin, along with the closely related iridoid glucoside, secologanin. CrNPF2.4, CrNPF2.5 and CrNPF2.6 were able to import both compounds (Fig. 1A). The three transporters grouped phylogenetically within the CrNPF2 subclade (Supplementary Fig. S2). The remaining five transporters were unable to import loganin and secologanin, and were pooled to serve as a negative control in subsequent experiments (this pool of transporters is referred to as the CrNPF2 pool).

### Biochemical characterization of *C. roseus* transporters

**Fig. 2:**
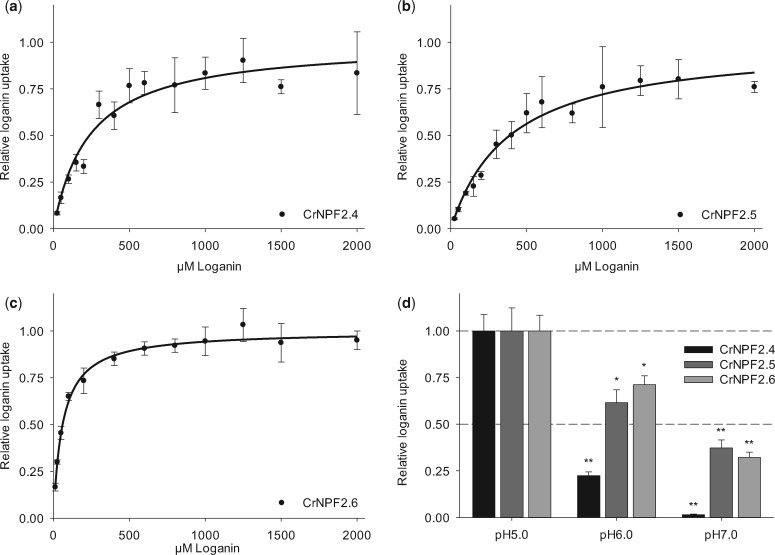
Kinetic characterization of CrNPF2.6, CrNPF2.4 and CrNPF2.5. (A–C) Michaelis–Menten saturation curves for loganin uptake for (A) CrNPF2.4, (B) CrNPF2.5 and (C) CrNPF2.6 [error bars are the SE; *n* = 4 (4 X 4 oocytes) for all data points]. For the relative loganin uptake in (A–C), *V*_max_ is defined as 1. (D) CrNPF2.6, CrNPF2.4 and CrNPF2.5 pH dependency for loganin transport. The relative loganin uptake displayed on the *y-*axis in (D) is the loganin uptake rate at the given pH normalized to the uptake rate of each of the respective transporters at pH 5.0 [error bars are the SE; *n* = 3 (3 X 3 oocytes) for all data points; *P < 0.05, **P < 0.01, Student’s *t*-test]. Loganin contents (A–D) were quantified by LC-MS analysis.

To provide insight into the physiological role of CrNPF2.4, CrNPF2.5 and CrNPF2.6, substrate specificities were investigated in *Xenopus* oocytes by measuring uptake activities of four intermediates in the seco-iridoid pathway: 7-deoxyloganic acid, loganic acid, loganin and secologanin. CrNPF2.4, CrNPF2.5 and CrNPF2.6 transported all substrates (Fig. 1B), but appeared to exhibit only low relative transport activity towards loganic acid. This was unexpected as loganic acid is reasoned to be the most likely mobile intermediate between IPAP and epidermis cells (Miettinen et al*.* 2014). For each of the three transporters, kinetic studies were performed in *Xenopus* oocytes using loganin as the model substrate, due to its commercial availability. The Michaelis–Menten equation was fitted to the uptake data assuming single site saturation. The *K*_m_ values for loganin for CrNPF2.4, CrNPF2.5 and CrNPF2.6 were 237 ± 35, 387 ± 57 and 60 ± 5 µM, respectively, at pH 5.0 (Fig. 2A–C). This classifies CrNPF2.6 as a high-affinity transporter and CrNPF2.4 and CrNPF2.5 as medium affinity loganin transporters. Saturating conditions for transport were not achieved for CrNPF2.4 and CrNPF2.5.

NPF members are typically proton-dependent symporters (Nour-Eldin et al*.* 2012, Léran et al*.* 2014). We therefore investigated the pH dependency of CrNPF2.4, CrNPF2.5 and CrNPF2.6, by assaying for loganin uptake at pH 5.0, pH 6.0 and pH 7.0. All three transporters displayed decreased loganin transport activity with increasing pH (Fig. 2D). The pH dependency was most pronounced for CrNPF2.4, having <4% activity at pH 7.0 (compared with pH 5.0). For CrNPF2.6 and CrNPF2.5, activity decreased below 40% at pH 7.0. This strongly suggests that the transporters are proton-dependent symporters. Additionally, to verify that the transporters were not promiscuous glucose transporters, accepting iridoid glucosides as substrates, loganin transport was measured in the presence of glucose in 10-fold excess. For all three transporters, the loganin uptake activity was unaffected by the presence of glucose (Supplementary Fig. S3). The high-affinity CrNPF2.6 transporter was, furthermore, tested for ion dependency of loganin transport. No effect was observed in response to altered Na^+^ to K^+^ ratios (Supplementary Fig. S4). Finally, we tested whether the loganin import of CrNPF2.6 was due to active transport activity by observing if the substrate accumulated inside the transporter-expressing oocytes to concentrations above the media concentration. For CrNPF2.6, loganin accumulated to almost 3-fold the media concentration when exposed to 12.5 µM loganin for 20 min. This demonstrated that CrNPF2.6 actively transports loganin (Supplementary Fig. S5).

### Identification of *C. roseus* loganin export activity

**Fig. 3:**
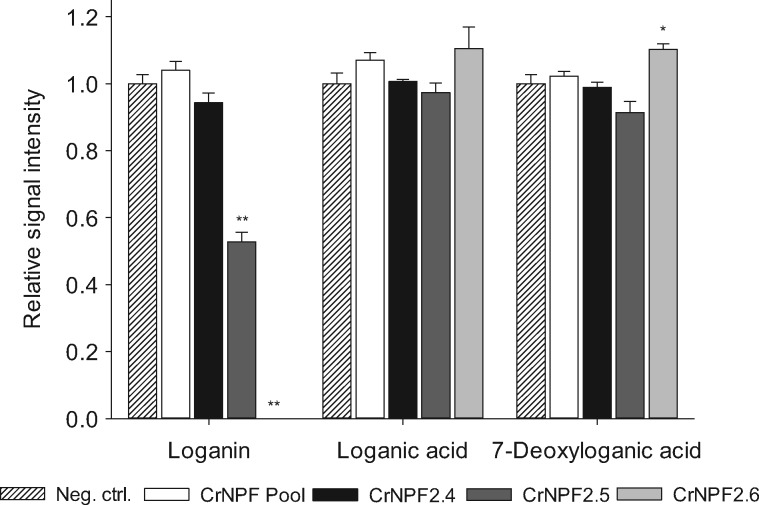
Iridoid glucoside efflux assay of the CrNPF2 transporters in *Xenopus* oocytes. The eight CrNPF2 transporters were screened for iridoid glucoside efflux from *Xenopus* oocytes by cRNA and substrate co-injection. After transporter expression, the internal loganin, loganic acid and 7-deoxyloganic acid concentrations in the oocytes were normalized to the substrate content of oocytes injected with a water and substrate mix (Neg. ctrl) and displayed as relative signal intensity [error bars are the SE; *n* = 3 (3 X 3 oocytes) for all data points; *P < 0.05, **P < 0.01, Student’s *t*-test]. Iridoid glucosides were detected by LC-MS.

Since the directionality of secondary active plant transporters is dependent on proton and substrate gradients (Geiger 2015), we investigated if reversing the gradients resulted in export activity. All eight *C. roseus* transporters were tested for export activity by co-injecting CrNPF2 cRNAs with a mixture of 7-deoxyloganic acid, loganic acid, loganin and secologanin into the oocytes. After incubation for 3 d (to allow for transporter expression), iridoid glucoside export was measured as a decrease in substrate content within the oocytes. Loganin was the only substrate that showed transporter-dependent decreases and only in CrNPF2.6- and CrNPF2.5-expressing oocytes (Fig. 3). Loganic acid and 7-deoxyloganic acid levels remained close to the levels of the CrNPF2 pool- and water-injected control oocytes for all transporters, indicating that they were not exported. Secologanin could not be detected in any of the injected oocytes. This suggests that secologanin was either metabolized within the oocyte or exported by endogenous transporters. Together, the import and export data demonstrated reversibility of the direction of loganin transport, but only for CrNPF2.5 and CrNPF2.6.

It is surprising that none of the transporters was able to export loganic acid and 7-deoxyloganic acid as proton-dependent transporters—at least theoretically—can transport bidirectionally, depending on the electrochemical gradients (Geiger 2015). A possible explanation could be that the oocyte cytosol functions as an acid trap. The different protonation states of the carboxylic acids, loganic acid and 7-deoxyloganic acid, at pH 7.4 inside the oocyte vs. pH 5.0 outside, may influence substrate recognition by the transporters.

### Characterization of CrNPF2.6 loganin export activity

**Fig. 4:**
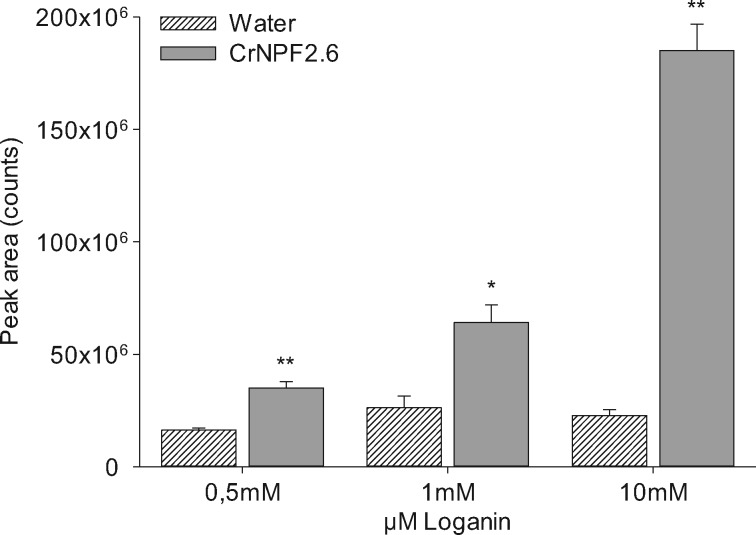
Loganin export by CrNPF2.6. Loganin export from CrNPF2.6-expressing oocytes injected to a final internal concentration of 0.5, 1 and 10 mM loganin. Loganin accumulation was detected in the media by LC-MS [error bars are the SE; *n* = 3 (3 X 3 oocytes) for all data points]. *t*-tests were performed between the media samples of oocytes not expressing heterologous transporters (Water) and media samples of the CrNPF2.6-expressing oocytes to confirm that the loganin transport was transporter dependent (*P < 0.05, **P < 0.01).

To investigate if loganin was truly exiting the oocytes in a transport-dependent manner, as opposed to being immobilized or metabolized, an export assay was developed to measure the loganin concentrations in the media outside CrNPF2.6-expressing oocytes. Loganin was injected directly into the transporter-expressing oocytes to achieve internal substrate concentrations of approximately 0.5, 1 and 10 mM. Loganin build-up in the medium revealed that export was facilitated by CrNPF2.6 in a concentration-dependent manner, confirming our results from the first export assay. The data also revealed a concentration-independent background level of loganin exported from the oocytes. It is possible that this intrinsic loganin export is the result of saturated endogenous transporter activity at all the tested substrate concentrations or leakage, post-substrate injection. Nevertheless, the CrNPF2.6-dependent export was significantly larger than the endogenous export for all tested loganin concentrations (Fig. 4). Comparison of import and export activity suggests that the transporter functions as an importer.

### Characterization of CrNPF2 localization and expression

**Fig. 5:**
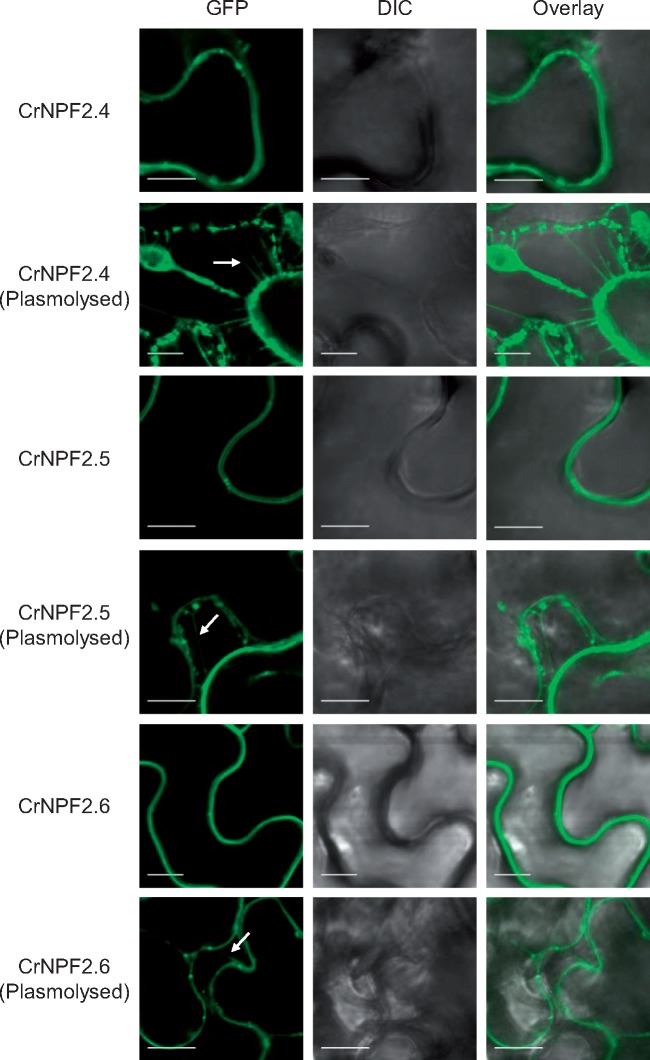
CrNPF2.4, CrNPF2.5 and CrNPF2.6 localize to the plasma membrane upon transient expression in *N. benthamiana.* GFP-tagged variants of CrNPF2.4, CrNPF2.5 and CrNPF2.6 were infiltrated into *N. benthamiana* leaves to investigate subcellular localization by confocal imaging. All three GFP-tagged CrNPFs were imaged before and after plasmolysis. Plasma membrane localization was evident for all CrNPFs by the formation of Hechtian strands upon plasmolysis. Arrows point to observed Hechtian strands. Differential interference contrast (DIC) microscopy images show cell morphology. Scale bars are 10 µm.

**Fig. 6:**
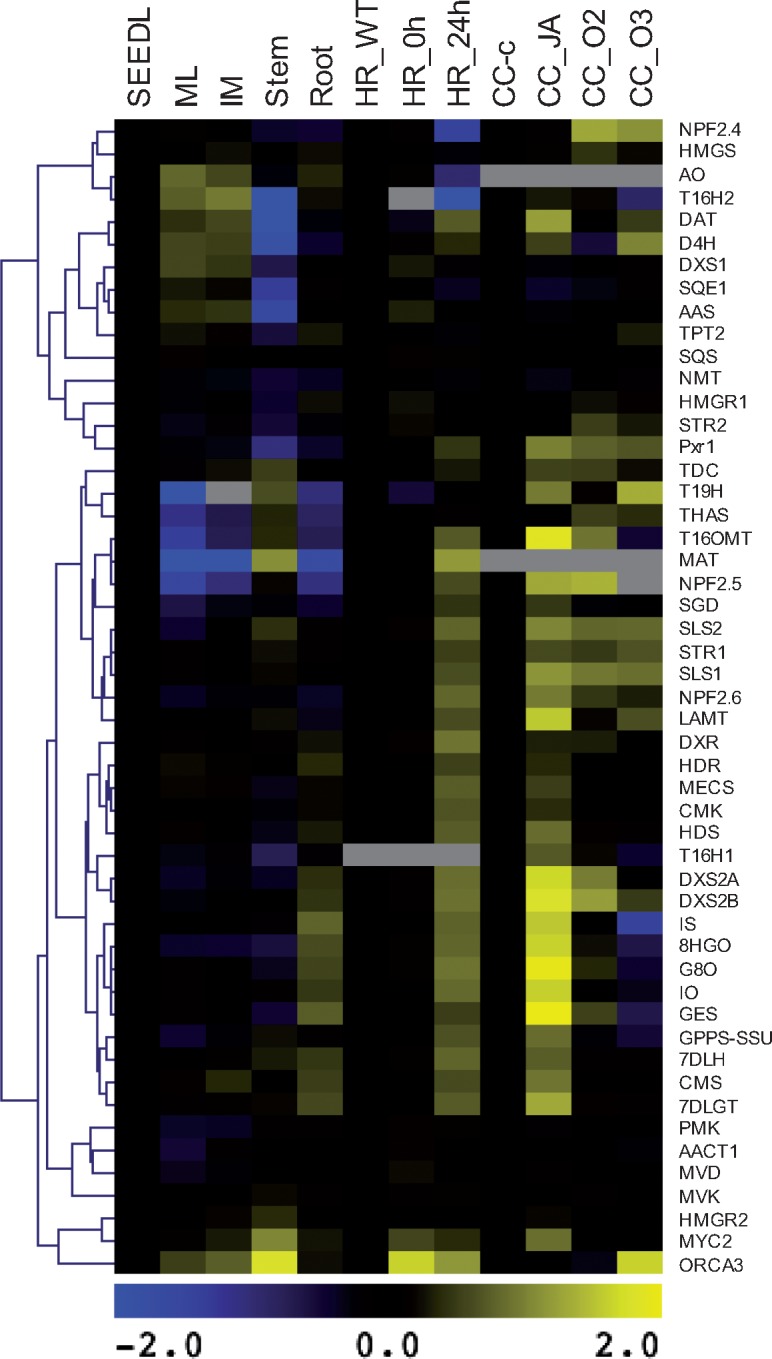
Co-expression analysis of *CrNPF* and terpenoid biosynthetic genes. The co-expression of *CrNPF2.4*, *CrNPF2.5* and *CrNPF2.6* with known terpenoid biosynthetic genes was assessed by cluster analysis of expression patterns using three compendia consisting of selected RNA-Seq data from (i) *C. roseus* organs from the MPGR consortium, in which values were normalized to the seedling reads (SEEDL) (ML, mature leaf; IM, immature leaf); (ii) *C. roseus* hairy roots from the MPGR consortium, in which values were normalized to wild-type hairy roots (HR_WT) (HR_6h and HR_24h were hairy roots treated with MeJA for 6 h and 24 h); and (iii) *C. roseus* cell suspension cultures from the ORCAE database from the SmartCell consortium (http://bioinformatics.psb.ugent.be/orcae/overview/Catro), in which values were normalized to the control cell culture (CC_c) (CC_JA, jasmonic acid-treated cell culture; CC_O2 and CC_O3, cell culture overexpressing *ORCA2* and *ORCA3*). Average linkage hierarchical clustering with Pearson correlation was used. Blue and yellow denote relative down-regulation and up-regulation to the corresponding control in each of the three compendia, respectively. Genes indicated in gray were not expressed in particular organs or cultures.

Previous studies have shown that NPF transporters localize either to the plasma membrane or to the tonoplast (Weichert et al*.* 2012). Subcellular localization of CrNPF2.4, CrNPF2.5 and CrNPF2.6 was therefore investigated by infiltrating *N. benthamiana* leaves with constructs encoding green fluorescent protein (GFP)-tagged variants of the transporters. Post-plasmolysis, confocal imaging of the infiltrated leaves shows all three transporters to be localized to the plasma membrane, seeing that Hechtian strands were formed (Fig. 5). We then investigated the expression levels of the transporter genes, *CrNPF2.4*, *CrNPF2.5* and *CrNPF2.6*, and all known characterized genes from the MIA, triterpene and their precursor pathways by mining publicly available *C. roseus* transcriptome data (Van Moerkercke et al*.* 2013). Co-regulation was assessed by hierarchical clustering of expression data from three RNA-Seq compendia: (i) selected *C. roseus* tissues; (ii) *C. roseus* hairy roots elicitated with jasmonate; and (iii) *C. roseus* suspension cells elicited with jasmonate or overexpressing transcription factors (Fig. 6). The data indicated that the three transporters were expressed in all tested *C. roseus* organs and were jasmonate inducible in seedlings. Expression of *CrNPF2.5* and *CrNPF2.6* was also jasmonate inducible in hairy roots and suspension cells (Fig. 6; Supplementary Table S4). Furthermore, the cluster analysis indicated that *CrNPF2.6*, encoding the transporter with the highest affinity for loganin, grouped together with *LAMT*, *SLS*, *strictosidine synthase* (*STR*) and *strictosidine glucosidase* (*SGD*) genes, and showed the highest co-regulation with these genes across the three compendia. *CrNPF2.6* expression is additionally controlled by the known MIA regulator ORCA (octadecanoid-responsive Catharanthus AP2-domain) (van der Fits and Memelink 2000, Miettinen et al*.* 2014), like the *SLS* and *STR* genes, further supporting its role in MIA synthesis (Fig. 6)*.*

**Fig. 7:**
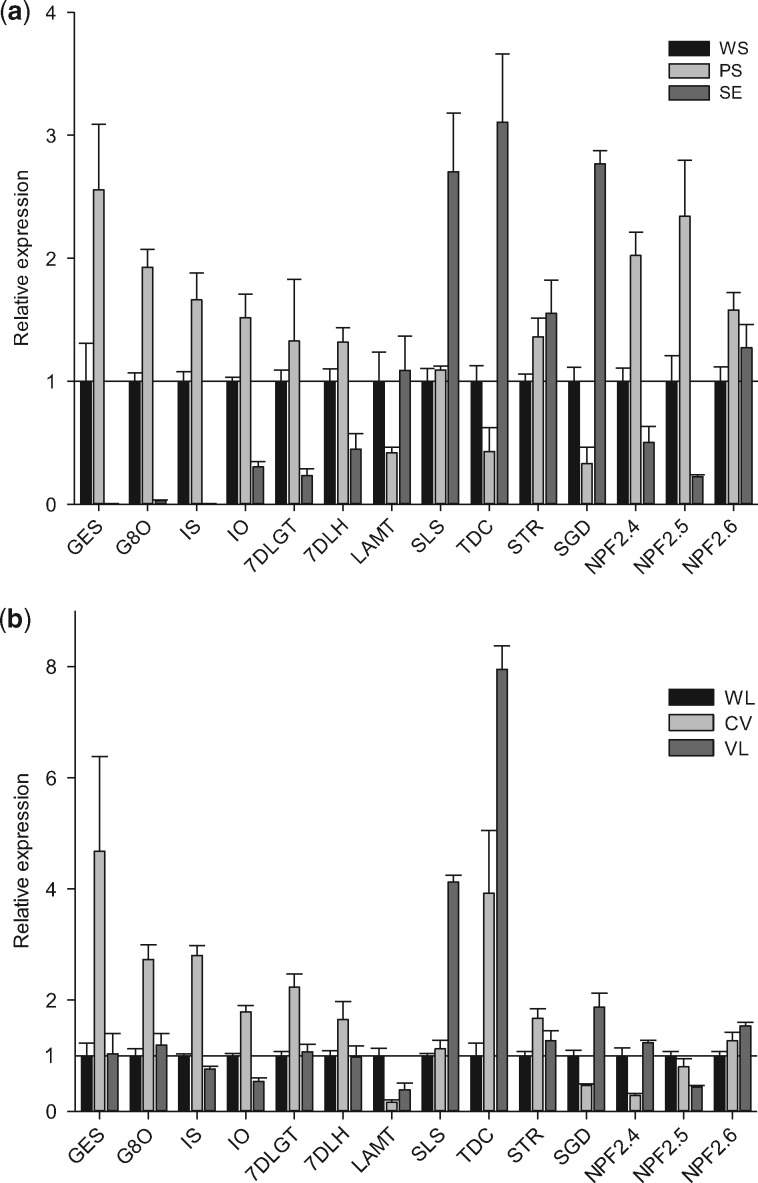
Expression of *CrNPF2.4*, *CrNPF2.5*, *CrNPF2.6* and MIA biosynthetic genes in *C. roseus* tissues. Quantitative PCR analysis showing expression of *CrNPF2.4*, *CrNPF2.5*, *CrNPF2.6* and MIA biosynthetic genes in stem- (A) and leaf-derived (B) tissues. Values are normalized to the controls, whole stems (A) and whole leaves (B), set to 1 (error bars are the standard error; *n* = 3 biological repeats). WS, whole stem; PS, peeled stem; SE, stem epidermis; WL, whole leaf; CV, central leaf vein; VL, veinless leaf tissue; GES, geraniol synthase; G8O, geraniol 8-oxidase; IS, iridoid synthase; IO, iridoid oxidase; 7DLGT, 7-deoxyloganetic acid glucosyltransferase; 7DLH, 7-deoxyloganic acid hydrolase; LAMT, loganate *O*-methyltransferase; SLS, secologanin synthase; TDC, l-tryptophan decarboxylase; STR, strictosidine synthase; SGD, strictosidine β-d-glucosidase.

Next, we investigated *CrNPF* expression at the cellular level. In situ hybridization was attempted on young seedling leaves, the material typically used for this technique. Unfortunately, no signals were obtained for *CrNPF2.4* or *CrNPF2.6*, the two transporters with the highest expression in this tissue (Supplementary Table S4). The lack of detectable signal showed that the level of *CrNPF2.4* and *CrNPF2.6* transcripts was low as compared with that of the enzyme-encoding genes (Supplementary Table S4). Therefore, we investigated the cell specificity of *CrNPF2.4*, *CrNPF2.5* and *CrNPF2.6* expression together with MIA pathway genes by quantitative real-time PCR (qPCR) on two sets of *C. roseus* tissues. The first set was derived from stems, from which we separated the epidermis from the rest of the stem tissue, and the second set was derived from leaves, from which we dissected the central vein, as well as nearly veinless tissue (Van Moerkercke et al*.* 2015). These sets were validated by expression analysis of known epidermal marker genes, such as *tryptophan decarboxylase* (*TDC*) and *SGD* (St-Pierre et al*.* 1999, Guirimand et al*.* 2010), and known IPAP-localized transcripts, such as *geraniol synthase* (*GES*), *geraniol-8-oxidase* (*G8O*) and *iridoid synthase* (*IS*) (Burlat et al*.* 2004, Simkin et al*.* 2013). This analysis did not reveal a pronounced cellular specificity in the expression of *CrNPF2.6* (Fig. 7), suggesting that this transporter may be expressed across different cell types, both in leaves and in stems. In the stem, *CrNPF2.4* and *CrNPF2.5* exhibited a similar expression pattern to the iridoid synthesis genes *iridoid oxidase* (*IO*)*, 7-deoxyloganetic acid glucosyl transferase* (*7DLGT*) and *7DLH*, i.e. all showed enrichment in non-epidermal cells, although not as absolute as the markedly IPAP-specific genes *GES*, *G8O* and *IS* (Fig. 7). In the leaves, *CrNPF2.4* exhibited a similar expression pattern to *LAMT*, for which we notably did not observe enrichment in the epidermis, either in stems or in leaves. This suggests that *LAMT* may also be expressed in non-epidermal cells, as also suggested by Murata et al*.* (2008). Together, this suggests that loganin production may not be restricted to the epidermis and that the CrNPF transporters could function as loganin importers in the epidermis. The enrichment of *7DLH* and *7DLGT* expression in IPAP tissues, particularly in stem tissue, also confirmed loganic acid as a candidate mobile intermediate. However, the IPAP enrichment of these two genes was less pronounced than that of the upstream iridoid genes, and expression of both genes was not excluded from epidermal-enriched tissue, as was the case for the upstream iridoid genes (Fig. 7). This analysis suggests 7-deoxyloganic acid as yet another candidate mobile intermediate. Conversely, although *SLS* expression was clearly enriched in epidermal tissues, it behaved differently from, for example, *SGD* as it was still present in IPAP-enriched tissues (Fig. 7). This suggests that secologanin may also be a candidate mobile intermediate.

## Discussion

By functional screening of an Arabidopsis transporter library in *Xenopus* oocytes, we initially identified the loganin-transporting AtNPF2.9, and, secondly, by screening eight orthologous *C. roseus* transporters, we identified three CrNPF2 transporters, CrNPF2.4, CrNPF2.5 and CrNPF2.6, capable of transporting the iridoid glucosides 7-deoxyloganic acid, loganic acid, loganin and secologanin. The three transporters localized to the plasma membrane and imported the four iridoid glucosides with different relative activity at pH 5.0, i.e. the pH of the apoplast. With loganin as substrate, we characterized CrNPF2.6 as a high-affinity iridoid glucoside transporter and CrNPF2.4 and CrNPF2.5 as medium affinity transporters.

In the seco-iridoid pathway, loganic acid is reasoned to be the mobile intermediate which is moved between IPAP and epidermal cells. This prediction is based on the expression of *7DLH* (produces loganic acid from 7-deoxyloganic acid) and *LAMT* (produces loganin from loganic acid) in IPAP and epidermis cells, respectively (Courdavault et al*.* 2014, Miettinen et al*.* 2014). The evidence for *7DLH* expression in IPAP cells comes from RNA in situ hybridization experiments in leaves, as well as proteomics analysis, that show enriched *7DLH* expression in mesophyll-derived protoplasts (which include the IPAP cells), compared with epidermis-derived protoplasts (Miettinen et al*.* 2014). Similarly, the evidence for *LAMT* expression in leaf epidermis cells is RNA in situ hybridization and proteomics data that show enrichment in leaf epidermis, compared with the whole leaf (Murata et al*.* 2008, Guirimand et al*.* 2011). *LAMT* expression is, however, not restricted to epidermal cells. *LAMT* may also be expressed in other cell types (Murata et al*.* 2008). Interestingly, LAMT is not the only pathway enzyme being expressed across several tissues. According to our qPCR data, pathway genes including *7DLGT*, *7DLH*, *LAMT* and *SLS* clearly show expression in additional cell types to those previously reported. This strongly suggests that there is a significant overlap in expression of pathway enzymes between IPAP and epidermal cells.

The high affinity of CrNPF2.6 for loganin combined with *LAMT* expression across several tissues suggests that loganin may also be a mobile intermediate. However, although LAMT is highly specific for loganic acid, the *K*_m_ value of 12.5–14.8 mM is remarkably high (Madyastha et al*.* 1973, Murata et al*.* 2008) and >1,000-fold higher than those of two previously characterized carboxyl methyltransferases (jasmonic acid methyltransferase and salicylic acid methyltransferase) (Ross et al*.* 1999, Seo et al*.* 2001). Thus, one may speculate whether another, as yet unidentified, methyltransferase, located within the IPAP cells, could be responsible for synthesizing loganin from loganic acid, supporting that loganin is the predominant mobile intermediate. Mining of the *C. roseus* transcriptome did not, however, yield any obvious candidate methyltransferase homolog with high sequence similarity or relevant co-expression pattern.

Our identification of three iridoid glucoside transporters that transport multiple pathway intermediates (7-deoxyloganic acid, loganin, secologanin as well as loganic acid) challenges the pathway model with only one mobile pathway intermediate, loganic acid. The simultaneous expression of *7DLGT*, *7DLH*, *LAMT* and *SLS in particular* in both IPAP and epidermal cells suggests a functional overlap in the pathway between the two cell types. Noticeably, in our expression analysis, a gradual increase of *IO*, *7DLGT*, *7DLH*, *LAMT* and *SLS* expression was observed in the epidermis of *C. roseus* stems. This suggests that these enzymes are expressed along a gradient across the cell types and that different intermediates could be transported by one or more transporters (Fig. 7). Attempts to provide in planta evidence by down-regulation of the transporters by virus-induced gene silencing were, unfortunately, inconclusive (Supplementary Fig. S6). Respectable levels of gene silencing, between 42% and 71%, were achieved for *CrNPF2.4* and *CrNPF2.6*, individually and for the two genes in combination. This did, however, not produce a metabolic phenotype. We expect that the redundancy in transport activity will require simultaneous, complete silencing, or gene knockout, of all three CrNPF transporters before a phenotype can be observed.

Our approach, screening a sequence-indexed transporter library from a heterogeneous species, enabled the identification of three iridoid glucoside transporters from *C. roseus*, although unknown transporters with less promiscuous substrate recognition profiles and higher affinity may also exist. Recent studies on transport substrate specificity have shown that transporters within a given species can be rather promiscuous and capable of transporting compounds foreign to its host. As an example, the similar but structurally distinct cyanogenic glucosides and glucosinolates can be transported by the NPF transporter Me14G074000 from *Manihot esculenta* (cassava), although glucosinolates are not synthesized by this species (JØrgensen et al*.* 2017).

**Fig. 8:**
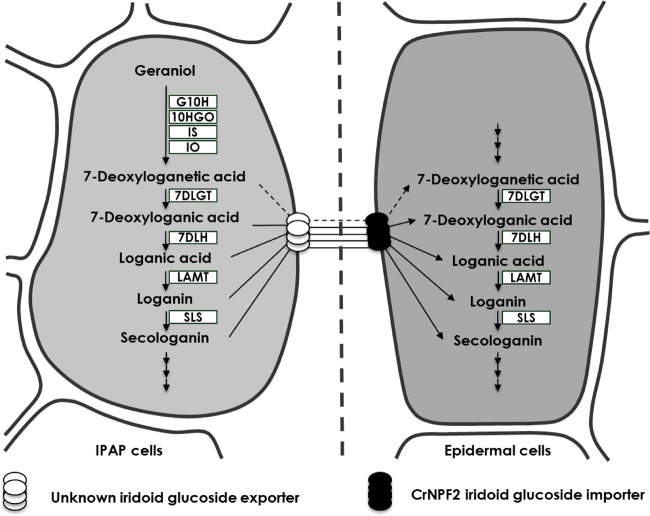
Model for orchestration of the seco-iridoid pathway with multiple mobile intermediates. The broad iridoid glucoside substrate specificity of CrNPF2.4, CrNPF2.5 and CrNPF2.6 allows for the existence of multiple mobile intermediates between the IPAP and epidermal cells. This suggests that one or more of the three transporters could function to import mobile intermediates into epidermal cells or other cell types where MIAs are produced.

The identification of three CrNPF transporters capable of importing four different iridoid glucosides supports the possibility of having multiple mobile pathway intermediates and we therefore propose that CrNPF2.4, CrNPF2.5 and CrNPF2.6 play a role in transporting multiple iridoid intermediates between IPAP and epidermal cells (Fig. 8). We will continue rigorously to resolve the localization of pathway intermediates, biosynthetic enzymes and transporters across all relevant plant tissues, to refine our understanding of the complex orchestration and organization of this model pathway.

## Materials and Methods

### Functional screening of an Arabidopsis transporter cDNA library expressed in *Xenopus* oocytes

A library consisting of 290 full-length Arabidopsis transporter cDNAs was screened for loganin uptake in *Xenopus* oocytes. The screen was performed using the transporter identification platform developed by Nour-Eldin et al*.* (2006, 2012). In brief, the library was divided into 29 pools of each 10 cRNA species (50 ng µl^–1^ cRNA) and was injected into *Xenopus* oocytes. Injected oocytes were kept for 2–4 d at 17°C in Kulori pH 7.4 (90 mM NaCl, 1 m M KCl, 1 mM CaCl_2_, 1 mM MgCl_2_ and 5 mM HEPES), containing 100 µg ml^–1^ gentamycin (Duchefa Biochemie). Loganin uptake assays were performed by incubating transporter-expressing oocytes for 1 h in Kulori pH 5.0 (90 mM NaCl, 1 mM KCl, 1 mM CaCl_2_, 1 mM MgCl_2_ and 5 mM MES) with 0.5–1 mM loganin {(1S,4aS,6S,7R,7aS)-6-hydroxy-7-methyl-1-[(2S,3R,4S,5S,6R)-3,4,5-trihydroxy-6-(hydroxymethyl)oxan-2-yl]oxy-1,4a,5,6,7,7a-hexahydrocyclopenta[c]pyran-4-carboxylate; Sigma}. Methanol extracts were prepared from the assayed oocytes by disrupting them in 50% (v/v) methanol. After overnight incubation at –20°C, the extracts were centrifuged (>14,000 relative centrifugal force at 4°C for 10 min) and the supernatants were analyzed by liquid chromatography–mass spectrometry (LC-MS) (described below). Pools testing positive for loganin uptake were assayed as single transporters, to identify the membrane protein(s) responsible for transport.

### Identification of the CrNPFs in *C. roseus*

By performing BLASTX searches in the CathaCyc database [a metabolic pathway database from *C. roseus* built from RNA-Seq data (Van Moerkercke et al*.* 2013) (www.cathacyc.org)] using the nucleotide sequence of AtNPF2.9 (AT1G18880) and CRG200 (Genbank accession AM232415, corresponding to CrNPF2.1; Caros007724.1) (Rischer et al*.* 2006) as query, transcripts corresponding to at least 40 NPF proteins were identified. To obtain the full-length open reading frames of partial sequences, additional BLASTN searches with the partial sequences were performed in CathaCyc and the *C. roseus* transcriptome database from the Medicinal Plant Genomics Resource (MPGR) consortium (medicinalplantgenomics.msu.edu).

### Cloning of CrNPF transporter genes

For heterologous expression in *Xenopus* oocytes, CrNPF2.1 (Caros 007724.1), CrNPF2.7 (Caros010326.1), CrNPF2.6 (Caros010208.1), CrNPF2.8 (Caros019517.1), CrNPF2.4 (Caros022254.1), CrNPF2.3 (Caros027020.1), CrNPF2.2 (Caros028411.1) and CrNPF2.5 (Caros015290.1) were Gateway cloned from *C. roseus* cDNA into pDONR221 (Invitrogen) and subcloned into the oocyte expression vector pOO2-GW (Ludewig et al*.* 2002, Chen et al*.* 2010). RNA was isolated from methyljasmonate (MeJA)-treated *C. roseus* leaves using the RNeasy plant mini kit (Qiagen) and cDNA was synthesized with the iScript cDNA kit (BioRad). The transporters were PCR amplified from the cDNA using primers containing attB sites (Supplementary Table S1). PCR amplification was performed with the Phusion High-Fidelity DNA Polymerase (Thermo Scientific) according to the manufacturer’s instructions. PCR products were purified using the GeneJet Gel Extraction Kit (Thermo Scientific), BP recombined into pDONR221 (Invitrogen) according to manufacturer’s instructions and transformed into chemically competent *Escherichia coli* DH5α cells. Entry plasmids were isolated using the GeneJet plasmid miniprep kit (Thermo Scientific) according to the manufacturer’s instructions [using kanamycin (Duchefa Biochemie) for selection], sequence verified, and LR recombined into pOO2-GW. LR reactions were performed using 1 µl of pOO2-GW (∼50 ng µl^–^^1^), 1 µl of Entry clone (∼70 ng µl^–1^) and 0.5 µl of LR clonase II (Invitrogen). The expression plasmids were isolated as described for the Entry clones.

### Preparation of cRNA for expression in *Xenopus* oocytes

The Entry plasmids were used to create linear DNA templates for in vitro transcription by PCR. PCR amplification was performed using the forward primer 5′TGTGCTGAATTGTAATACGACTCACTATAGGGAGCTTGCTTGTTCTTTTTGC3′, the reverse primer 5′CCATTCGCCATTCAGGCT3′ and HotMaster Taq DNA Polymerase (Five Prime), according to the manufacturer’s instructions. The following PCR amplification cycle was used: initial denaturation at 94°C for 2 min; 35 cycles of 94°C for 20 s, 55°C for 10 s and 70°C for 2 min; final extension at 70°C for 10 min. PCR products were purified using the QIAquick PCR Purification Kits (Qiagen). In vitro transcription was performed for each transporter gene by mixing 8 µl of purified PCR product with 42 µl of transcription master mix: [1 X T7 transcription buffer (Fermentas), 10 mM dithiothreitol (DTT), 25 µg ml^–1^ bovine serum albumin (BSA), 1 mM rATP, 1 mM rUTP, 1 mM rCTP, 0.05 mM rGTP (Illumina), 80 U of T7 RNA polymerase (Fermentas), 20 U of Ribolock RNase (Fermentas), 0.01 U of inorganic pyrophosphatase (Fermentas) and 0.06 U of 3′-OMe-7 mG(5′)ppp(5′)G RNA cap structure analog (NEB)]. The reactions were incubated at 37°C for 30 min (capping step) before 0.5 µl of 100 mM rGTP was added. Incubation was continued at 37°C for an additional 2–3 h. The cRNAs were recovered by LiCl precipitation. Briefly, 100 µl of 7.5 M LiCl was added to each reaction before storing them overnight at –20°C. The cRNAs were pelleted by centrifugation (>14,000 relative centrifugal force for 15 min at 4°C) and the supernatants were discarded. The pellets were washed with 70% (v/v) ethanol and air-dried. The cRNAs were resuspended in 20 µl of water and concentrations were normalized to 200 ng µl^–1^.

### Xenopus oocyte preparation

*Xenopus* oocytes were ordered from Ecocyte Bioscience or prepared as described by Romero et al*.* (1998). Briefly, oocytes were obtained by surgery, dissected and defolliculated for 1–2 h, with horizontal mixing (60 r.p.m.), in digestion solution [90 mM NaCl, 1 mM KCl, 1 mM CaCl_2_, 1 mM MgCl_2_, 5 mM HEPES, 10 g l^–1^ collagenase type 1 (Worthington) and 1 g l^–1^ trypsin inhibitor (Sigma)]. Oocytes were washed (90 mM NaCl, 1 mM KCl, 1 mM CaCl_2_, 1 mM MgCl_2_, 5 mM HEPES and 1 g l^–1^ BSA) and incubated for 1 h in phosphate buffer pH 6.5 (1 g l^–1^ BSA and 100 mM K_2_HPO_4_). After a final wash-step with Kulori buffer pH 7.4, stage V and VI oocytes were selected for injection on the following day.

### Transporter expression in *Xenopus* oocytes by cRNA microinjection

For transporter expression, *Xenopus* oocytes were injected with single transporter cRNAs (200 ng µl^–1^) or the CrNPF2 pool (CrNPF2.1, CrNPF2.2, CrNPF2.3, CrNPF2.7 and CrNPF2.8; 40 ng µl^–1^ of each cRNA). Mock RNA or water was used to inject oocytes which served as negative controls. The cRNAs were manually injected into oocytes using a Nanoject II™ Auto-Nanoliter Injector (Drummond) set to inject 50 nl. After injection, the oocytes were incubated for 3 d at 17°C in Kulori pH 7.4 with 100 µg ml^–1^ gentamycin.

### Uptake assays

Uptake assays (not including uptake assays for *K*_m_ determination; see below) were performed by incubating transporter-expressing oocytes for 30–60 min at room temperature in Kulori pH 5.0-based buffers containing 250 µM 7-deoxyloganic acid {(1S,4aS,7S,7aR)-1-(β-d-glucopyranosyloxy)-7-methyl-1,4a,5,6,7,7a-hexahydrocyclopenta[c]pyran-4-carboxylic acid; provided by Karel Miettinen}, loganic acid {(1S,4aS,6S,7R,7aS)-1-(β-d-glucopyranosyloxy)-6-hydroxy-7-methyl-1,4a,5,6,7,7a-hexahydrocyclopenta[c]pyran-4-carboxylic acid; provided by Nicolas Navrot}, loganin or secologanin [methyl (2S,3R,4S)-3-ethenyl-2-(β-d-glucopyranosyloxy)-4-(2-oxoethyl)-3,4-dihydro-2H-pyran-5-carboxylate; Sigma]. Assays were stopped by rinsing the oocytes four times in Kulori pH 7.4 and disrupting them in 100 µl of 50% methanol. Samples were filtered using MSFBN6B MultiScreenHTS FB filter plates (1.0/0.65 µm, Milipore) and analyzed by LC-MS.

### *K*_m_ studies of loganin uptake

The *K*_m_ determination studies for CrNPF2.4, CrNPF2.5 and CrNPF2.6, using loganin, were performed at pH 5.0 in Kulori buffer using substrate concentrations ranging from 12.5 µM to 2 mM. Appropriate incubation times were determined for each substrate concentration, for all three transporters. This was done using uptake assays to identify incubation times for which the transport rate (*V*) was approximately equal to the initial transport velocity (*V*_0_). The purpose of these experiments was to define assay conditions where back-transport of substrate could be disregarded. All *K*_m_ assays were performed in media volumes of 500 µl with a minimum of 4 X 4 oocytes per substrate concentration. Oocyte extracts and LC-MS analysis were performed as previously described. The data was fitted to the Michaelis–Menten equation, assuming one site saturation {*f* = *B*_max_ X abs(*x*)/[*K*_d_ + abs(*x*)]} using SigmaPlot 12.5 (Systat Software).

### Efflux assays

For the export screen, the cRNAs were mixed with 7-deoxyloganic acid, loganic acid, loganin and secologanin, to a final concentration of 40 µM iridoid glucoside, prior to oocyte injection. The oocytes were incubated for 3 d at 17°C in Kulori pH 7.4 with 100 µg ml^–1^ gentamycin for transporter expression. On day 3, oocytes pools, injected with single cRNAs or cRNA pools, were split in two and incubated in Kulori pH 5.0 or pH 7.4 for 1h. Oocyte extracts were prepared for LC-MS analysis as described above for the uptake assays. Loganin export was further characterized for CrNPF2.6 using an additional export assay. CrNPF2.6-expressing oocytes were injected with loganin stock solutions to achieve internal concentrations of approximately 0.5, 1 and 10 mM (oocyte volume was assumed to be 1 µl). After a 10 min recovery period in Kulori buffer pH 7.5, the oocytes were transferred to Kulori pH 5.0 for 1 h. The loganin content in the Kulori pH 5.0 assay media was analyzed for its loganin content by LC-MS after filtering (1 µm).

### Iridoid glucoside detection by LC-MS analysis

LC-MS analysis was performed using an Agilent 1100 Series LC (Agilent Technologies) coupled to a Bruker HCT-Ultra ion trap mass spectrometer (Bruker Daltonics). The mass spectrometer was run in positive electrospray mode and loganin was detected from integration of extracted ion chromatograms. All iridoid glucosides were detected as single-charged sodium adducts [M + Na^+^]: 7-deoxyloganic acid, *m/z* 383; loganic acid, *m/z* 399; secologanin, *m/z* 411; and loganin, *m/z* 413. For details on the LC set-up, see Supplementary Table S2.

### Confocal microscopy of *Agrobacterium*-infiltrated *N. benthamiana* leaves

The constructs for CrNPF localization, and a *pCaMV35S:p19* construct to suppress gene silencing (Voinnet et al*.* 2003), were individually transformed into the *Agrobacterium tumefaciens* strain C58C1, carrying the pMP90 helper plasmid. The resulting strains were used for infiltration of *N. benthamiana* (Onrubia et al*.* 2014). Plasmolysis was performed by immersing leaf discs in 1 M KNO_3_ 10 min prior to imaging (Szydlowski et al*.* 2013). Microscopic analysis was carried out with an LSM 710 confocal laser scanning microscope (Zeiss) using a X 63 water immersion objective (numerical aperture of 1.2). GFP was excited at a wavelength of 488 nm and emission was detected at 500–550 m.

### Quantitative (q)PCR analysis of *C. roseus* stem and leaf tissues

Stem and leaf tissues were generated as described (Van Moerkercke et al*.* 2015). In brief, whole stem tissue was collected between the mature leaves of greenhouse-grown plants. Stem epidermis-enriched tissue and peeled stems were obtained by peeling mature stems with a potato peeler. Central leaf vein tissue was cut out from leaves with a scalpel. To obtain veinless leaf tissue from leaves, the central vein was removed from the leaf, and then the tissue between the secondary veins was cut out with a scalpel. Tissue samples were ground in liquid nitrogen and total RNA was extracted with RNeasy (Qiagen). A 1 µg aliquot of DNase-treated total RNA was used for cDNA synthesis with iScript (BioRad). Gene-specific primers for qPCR were designed with the online software Primer3 (http://biotools.umassmed.edu/bioapps/primer3_www.cgi) (Supplementary Table S3). Two reference genes for normalization, *N2227* and *SAND*, were used for the experiments (Pollier et al*.* 2014). qPCR was performed using a Lightcycler 480 (Roche) with SYBR Green QPCR master Mix (Stratagene). All measurements represent the average of three biological replicates; each biological replicate comprises two technical replicates.

### Data deposition

The sequences reported herein have been deposited in the GenBank data libraries under accession numbers KR054375–KR054382 for CrNPF2.1–CrNPF2.8, respectively.

## Supplementary data

Supplementary data are available at PCP online.

## Funding

This work was supported by the European Union Seventh Framework Programme FP7/2007–2013 [222716-SMARTCELL]; The Short-Term Scientific Missions (STSM) program from the European Union COST Action [FA1006-PlantEngine]; the European Molecular Biology Organization [Long-Term Fellowship to A.V.M.]; European Commission support from Marie Curie Actions [EMBOCOFUND2010 to A.V.M., GA-2010-267154 to A.V.M.]; the Research Foundation Flanders [G005212N to J.P., post-doctoral fellowship to J.P.]; the Swiss National Foundation [Early Postdoc Mobility grant to F.S. and M.C.]; the John Innes Centre [studentship to R.P.]; and The Danish National Research Foundation [DNRF99 to B.L. and B.A.H.].

## Supplementary Material

Supplementary Data

## Abbreviations

BSA: bovine seum albumin
7DLGT: 7-deoxyloganetic acid glucosyl transferase
7DLH: 7-deoxyloganic acid hydroxylase
GES: geraniol synthase
GFP: green fluorescent protein
G8O: geraniol-8-oxidase
IO: iridoid oxidase
IPAP: internal phloem-associated parenchyma
IS: iridoid synthase
LAMT: loganic acid methyltransferase
LC-MS: liquid chromatography–mass spectrometry
MIA, monoterpenoid indole alkaloid MPGR: Medicinal Plant Genomics Resource
NPF: NRT1/PTR family
qPCR: quantitative real-time PCR
SGD: strictosidine glucosidase
SLS: secologanin synthase
STR: strictosidine synthase
TDC: tryptophan decarboxylase

## References

[pcx097-B1] Brown S., Clastre M., Courdavault V., O’Connor S.E. (2015) De novo production of the plant-derived alkaloid strictosidine in yeast. Proc. Natl. Acad. Sci. USA 112: 3205–3210. 25675512 10.1073/pnas.1423555112 PMC4371906

[pcx097-B2] Burlat V., Oudin A., Courtois M., Rideau M., St-Pierre B. (2004) Co-expression of three MEP pathway genes and geraniol 10-hydroxylase in internal phloem parenchyma of *Catharanthus roseus* implicates multicellular translocation of intermediates during the biosynthesis of monoterpene indole alkaloids and isoprenoid-derived primary metabolites. Plant J. 38: 131–141. 15053766 10.1111/j.1365-313X.2004.02030.x

[pcx097-B3] Carqueijeiro I., Noronha H., Duarte P., GerÓs H., Sottomayor M. (2013) Vacuolar transport of the medicinal alkaloids from *Catharanthus roseus* is mediated by a proton-driven antiport. Plant Physiol. 162: 1486–1496. 23686419 10.1104/pp.113.220558 PMC3707533

[pcx097-B4] Chen L.-Q., Hou B.-H., Lalonde S., Takanaga H., Hartung M.L., Qu X.Q. (2010) Sugar transporters for intercellular exchange and nutrition of pathogens. Nature 468: 527–532. 21107422 10.1038/nature09606 PMC3000469

[pcx097-B5] Courdavault V., Papon N., Clastre M., Giglioli-Guivarc’h N., St-Pierre B., Burlat V. (2014) A look inside an alkaloid multisite plant: the Catharanthus logistics. Curr. Opin. Plant Biol. 19: 43–50. 24727073 10.1016/j.pbi.2014.03.010

[pcx097-B6] De Luca V., Salim V., Thamm A., Atsumi Masada S., Yu F. (2014) Making iridoids/secoiridoids and monoterpenoid indole alkaloids: progress on pathway elucidation. Curr. Opin. Plant Biol. 19: 35–42. 24709280 10.1016/j.pbi.2014.03.006

[pcx097-B7] Dugé de Bernonville T., Clastre M., Besseau S., Oudin A., Burlat V., Glévarec G. (2015) Phytochemical genomics of the Madagascar periwinkle: unravelling the last twists of the alkaloid engine. Phytochemistry 113: 9–23. 25146650 10.1016/j.phytochem.2014.07.023

[pcx097-B8] Geiger D. (2015) Plant sucrose transporters from a biophysical point of view. Mol. Plant. 4: 395–406. 10.1093/mp/ssr029 21502662

[pcx097-B9] Geu-Flores F., Sherden N.H., Courdavault V., Burlat V., Glenn W.S., Wu C. (2012) An alternative route to cyclic terpenes by reductive cyclization in iridoid biosynthesis. Nature 492: 138–142. 23172143 10.1038/nature11692 PMC13215347

[pcx097-B10] GÓngora-Castillo E., Childs K.L., Fedewa G., Hamilton J.P., Liscombe D.K., Magallanes-Lundback M. (2012) Development of transcriptomic resources for interrogating the biosynthesis of monoterpene indole alkaloids in medicinal plant species. PLoS One 7: e52506 23300689 10.1371/journal.pone.0052506 PMC3530497

[pcx097-B11] Guirimand G., Courdavault V., Lanoue A., Mahroug S., Guihur A., Blanc N. (2010) Strictosidine activation in Apocynaceae: towards a ‘nuclear time bomb’? BMC Plant Biol. 10: 182. 20723215 10.1186/1471-2229-10-182 PMC3095312

[pcx097-B12] Guirimand G., Guihur A., Ginis O., Poutrain P., Héricourt F., Oudin A. (2011) The subcellular organization of strictosidine biosynthesis in *Catharanthus roseus* epidermis highlights several trans-tonoplast translocations of intermediate metabolites. FEBS J. 278: 749–763. 21205206 10.1111/j.1742-4658.2010.07994.x

[pcx097-B13] Hildreth S.B., Gehman E.A., Yang H., Lu R.-H., Riresh K.C., Harich K.C. (2011) Tobacco nicotine uptake permease (NUP1) affects alkaloid metabolism. Proc. Natl. Acad. Sci. USA 108: 18179–18184. 22006310 10.1073/pnas.1108620108 PMC3207657

[pcx097-B14] Irmler S., SchrÖder G., St-Pierre B., Crouch N.P., Hotze M., Schmidt J. (2000) Indole alkaloid biosynthesis in *Catharanthus roseus*: new enzyme activities and identification of cytochrome P450 CYP72A1 as secologanin synthase. Plant J. 24: 797–804. 11135113 10.1046/j.1365-313x.2000.00922.x

[pcx097-B15] JØrgensen M., Xu D., Crocoll C., Ramírez D., Motawia M., Olsen C. (2017) Origin and evolution of transporter substrate specificity within the NPF family. eLife 6: 1–30. 10.7554/eLife.19466 PMC5336358 28257001

[pcx097-B16] Kidd S.K., Melillo A.A., Lu R.-H., Reed D.G., Kuno N., Uchida K. (2006) The A and B loci in tobacco regulate a network of stress response genes, few of which are associated with nicotine biosynthesis. Plant Mol. Biol. 60: 699–716. 16649107 10.1007/s11103-005-5546-z

[pcx097-B17] Larsen B., Xu D., Halkier B.A., Nour-Eldin H.H. (2017) Advances in methods for identification and characterization of plant transporter function. J. Exp. Bot. DOI:10.1093/jxb/erx140. 10.1093/jxb/erx140 28472492

[pcx097-B18] Léran S., Varala K., Boyer J.-C., Chiurazzi M., Crawford N., Daniel-Vedele F. (2014) A unified nomenclature of NITRATE TRANSPORTER 1/PEPTIDE TRANSPORTER family members in plants. Trends Plant Sci. 19: 5–9. 24055139 10.1016/j.tplants.2013.08.008

[pcx097-B19] Ludewig U., von Wirén N., Frommer W.B. (2002) Uniport of NH by the root hair plasma membrane ammonium transporter LeAMT1;1. J. Biol. Chem. 277: 13548–13555. 11821433 10.1074/jbc.M200739200

[pcx097-B20] Madyastha K.M., Guarnaccia R., Baxter C., Coscia C.J. (1973) S-Adenosyl-l-methionine: loganic acid methyltransferase. A carboxyl-alkylating enzyme from Vinca rosea. J. Biol. Chem. 248: 2497–2501. 4698228

[pcx097-B21] Mahroug S., Burlat V., St-Pierre B. (2007) Cellular and sub-cellular organisation of the monoterpenoid indole alkaloid pathway in *Catharanthus roseus**.* Phytochem. Rev. 6: 363–381.

[pcx097-B22] Miettinen K., Dong L., Navrot N., Schneider T., Burlat V., Pollier J. (2014) The seco-iridoid pathway from *Catharanthus roseus*. Nat. Commun. 5: 3606. 24710322 10.1038/ncomms4606 PMC3992524

[pcx097-B23] Morita M., Shitan N., Sawada K., Van Montagu M.C.E., Inzé D., Rischer H. (2009) Vacuolar transport of nicotine is mediated by a multidrug and toxic compound extrusion (MATE) transporter in *Nicotiana tabacum*. Proc. Natl. Acad. Sci. USA 106: 2447–2452. 19168636 10.1073/pnas.0812512106 PMC2650162

[pcx097-B24] Murata J., Roepke J., Gordon H., De Luca V. (2008) The leaf epidermome of *Catharanthus roseus* reveals its biochemical specialization. Plant Cell 20: 524–542. 18326827 10.1105/tpc.107.056630 PMC2329939

[pcx097-B25] Nour-Eldin H.H., Andersen T.G., Burow M., Madsen S.R., JØrgensen M.E., Olsen C.E. (2012) NRT/PTR transporters are essential for translocation of glucosinolate defence compounds to seeds. Nature 488: 531–534. 22864417 10.1038/nature11285

[pcx097-B26] Nour-Eldin H.H., Halkier B.A. (2013) The emerging field of transport engineering of plant specialized metabolites. Curr. Opin. Biotechnol. 24: 263–270. 23040969 10.1016/j.copbio.2012.09.006

[pcx097-B27] Nour-Eldin H.H., NØrholm M.H.H., Halkier B.A. (2006) Screening for plant transporter function by expressing a normalized Arabidopsis full-length cDNA library in Xenopus oocytes. Plant Methods 2: 17.7 17069646 10.1186/1746-4811-2-17 PMC1637106

[pcx097-B28] Onrubia M., Pollier J., Vanden Bossche R., Goethals M., Gevaert K., Moyano E. (2014) Taximin, a conserved plant-specific peptide is involved in the modulation of plant-specialized metabolism. Plant Biotechnol. J. 12: 971–983. 24852175 10.1111/pbi.12205

[pcx097-B29] Payne R.M., Xu D., Foureau E., Teto Carqueijeiro M.I.S., Oudin A., Bernonville T.D. (2017) An NPF transporter exports a central monoterpene indole alkaloid intermediate from the vacuole. Nat. Plants 3: 16208. 28085153 10.1038/nplants.2016.208 PMC5238941

[pcx097-B30] Pollier J., Bossche R., Vanden, Rischer H., Goossens A. (2014) Selection and validation of reference genes for transcript normalization in gene expression studies in *Catharanthus roseus*. Plant Physiol. Biochem. 83: 20–25. 25058454 10.1016/j.plaphy.2014.07.004

[pcx097-B31] Rischer H., Orešič M., Seppänen-Laakso T., Katajamaa M., Lammertyn F., Ardiles-Diaz W. (2006) Gene-to-metabolite networks for terpenoid indole alkaloid biosynthesis in *Catharanthus roseus* cells. Proc. Natl. Acad. Sci. USA 103: 5614–5619. 16565214 10.1073/pnas.0601027103 PMC1459402

[pcx097-B32] Romero M.F., Kanai Y., Gunshin H., Hediger M.A. (1998) Expression cloning using *Xenopus laevis* oocytes. Methods Enzymol. 296: 17–52. 9779438 10.1016/s0076-6879(98)96004-9

[pcx097-B33] Ross J.R., Nam K.H., D’Auria J.C., Pichersky E. (1999) S-Adenosyl-l-methionine:salicylic acid carboxyl methyltransferase, an enzyme involved in floral scent production and plant defense, represents a new class of plant methyltransferases. Arch. Biochem. Biophy. 367: 9–16. 10.1006/abbi.1999.1255 10375393

[pcx097-B34] Seo H.S., Song J.T., Cheong J.-J., Lee Y.-H., Lee Y.-W., Hwang I. (2001) Jasmonic acid carboxyl methyltransferase: a key enzyme for jasmonate-regulated plant responses. Proc. Natl. Acad. Sci. USA 98: 4788–4793. 11287667 10.1073/pnas.081557298 PMC31912

[pcx097-B35] Shitan N., Bazin I., Dan K., Obata K., Kigawa K., Ueda K. (2003) Involvement of CjMDR1, a plant multidrug-resistance-type ATP-binding cassette protein, in alkaloid transport in *Coptis japonica*. Proc. Natl. Acad. Sci. USA 100: 751–756. 12524452 10.1073/pnas.0134257100 PMC141068

[pcx097-B36] Shitan N., Dalmas F., Dan K., Kato N., Ueda K., Sato F. (2013) Characterization of *Coptis japonica* CjABCB2, an ATP-binding cassette protein involved in alkaloid transport. Phytochemistry 91: 109–116. 22410351 10.1016/j.phytochem.2012.02.012

[pcx097-B37] Shoji T., Inai K., Yazaki Y., Sato Y., Takase H., Shitan N. (2009) Multidrug and toxic compound extrusion-type transporters implicated in vacuolar sequestration of nicotine in tobacco roots. Plant Physiol. 149: 708–718. 19098091 10.1104/pp.108.132811 PMC2633862

[pcx097-B38] Simkin A.J., Miettinen K., Claudel P., Burlat V., Guirimand G., Courdavault V. (2013) Characterization of the plastidial geraniol synthase from Madagascar periwinkle which initiates the monoterpenoid branch of the alkaloid pathway in internal phloem associated parenchyma. Phytochemistry 85: 36–43. 23102596 10.1016/j.phytochem.2012.09.014

[pcx097-B39] St-Pierre B., Vazquez-Flota F.A., De Luca V. (1999) Multicellular compartmentation of *Catharanthus roseus* alkaloid biosynthesis predicts intercellular translocation of a pathway intermediate. Plant Cell 11: 887–900. 10330473 10.1105/tpc.11.5.887 PMC144229

[pcx097-B40] Szydlowski N., Bürkle L., Pourcel L., Moulin M., Stolz J., Fitzpatrick T.B. (2013) Recycling of pyridoxine (vitamin B6) by PUP1 in Arabidopsis. Plant J. 75: 40–52. 23551747 10.1111/tpj.12195

[pcx097-B41] van der Fits L., Memelink J. (2000) ORCA3, a jasmonate-responsive transcriptional regulator of plant primary and secondary metabolism. Science 289: 295–297. 10894776 10.1126/science.289.5477.295

[pcx097-B42] Van Moerkercke A., Fabris M., Pollier J., Baart G.J.E., Rombauts S., Hasnain G. (2013) CathaCyc, a metabolic pathway database built from *Catharanthus roseus* RNA-Seq data. Plant Cell Physiol. 54: 673–685. 23493402 10.1093/pcp/pct039

[pcx097-B43] Van Moerkercke A., Steensma P., Schweizer F., Pollier J., Gariboldi I., Payne R. (2015) The bHLH transcription factor BIS1 controls the iridoid branch of the monoterpenoid indole alkaloid pathway in *Catharanthus roseus*. Proc. Natl. Acad. Sci. USA 112: 8130–8135. 26080427 10.1073/pnas.1504951112 PMC4491741

[pcx097-B44] Verma P., Mathur A.K., Srivastava A., Mathur A. (2012) Emerging trends in research on spatial and temporal organization of terpenoid indole alkaloid pathway in *Catharanthus roseus*: a literature update. Protoplasma 249: 255–268. 21630129 10.1007/s00709-011-0291-4

[pcx097-B45] Voinnet O., Rivas S., Mestre P., Baulcombe D. (2003) An enhanced transient expression system in plants based on suppression of gene silencing by the p19 protein of tomato bushy stunt virus. Plant J. 33: 949–956. (Article has been retracted) 12609035 10.1046/j.1365-313x.2003.01676.x

[pcx097-B46] Weichert A., Brinkmann C., Komarova N.Y., Dietrich D., Thor K., Meier S. (2012) AtPTR4 and AtPTR6 are differentially expressed, tonoplast-localized members of the peptide transporter/nitrate transporter 1 (PTR/NRT1) family. Planta 235: 311–323. 21904872 10.1007/s00425-011-1508-7

[pcx097-B47] Yu F., De Luca V. (2013) ATP-binding cassette transporter controls leaf surface secretion of anticancer drug components in *Catharanthus roseus*. Proc. Natl. Acad. Sci. USA 110: 15830–15835. 24019465 10.1073/pnas.1307504110 PMC3785729

[pcx097-B100] Zürcher E., Liu J., di Donato M., Geisler M., Müller B.(2016) Plant development regulated by cytokinin sinks. Science 353: 1027–1030. 27701112 10.1126/science.aaf7254

