# Supplementary material for: Identification of Iridoid Glucoside Transporters in Catharanthus roseus
Source: Plant Cell Physiol. 2017 Jul 19;58(9):1507–18. doi: 10.1093/pcp/pcx097 (PMC5921532; doi:10.1093/pcp/pcx097)
Supplement: Supplementary Data [file pcx097_supplementary_pcp-2017-e-00194-file015.docx]

Supplementary Information

Supplementary Tables

| Supplementary Table 1 \| **Primer sequences for PCR amplifying the CrNPF2s.** | |
| --- | --- |
| **Primers** | **Primer sequence (5’ 🡪 3’)** |
| CrNPF2.1-ExtFW | GGGGACAAGTTTGTACAAAAAAGCAGGCTTAATGAAGAAGCAGCTGAGTTC |
| CrNPF2.1-ExtRV | GGGGACCACTTTGTACAAGAAAGCTGGGTATCAGTGTTTAAGATCATC |
| CrNPF2.6-ExtFW | GGGGACAAGTTTGTACAAAAAAGCAGGCTTAATGGAGAAAAACGAGAAATTAG |
| CrNPF2.6-ExtRV | GGGGACCACTTTGTACAAGAAAGCTGGGTATTAAACTAGATGCTTTTC |
| CrNPF2.7-ExtFW | GGGGACAAGTTTGTACAAAAAAGCAGGCTTAATGGAGAAAAAAGAGACC |
| CrNPF2.7-ExtRV | GGGGACCACTTTGTACAAGAAAGCTGGGTATCAAACAATAGCTTTCTC |
| CrNPF2.8-ExtFW | GGGGACAAGTTTGTACAAAAAAGCAGGCTTAATGGAGAAAAACCAGCAGAAG |
| CrNPF2.8-ExtRV | GGGGACCACTTTGTACAAGAAAGCTGGGTATCAAACAATGGCTTTATCTGTCTTT |
| CrNPF2.4-ExtFW | GGGGACAAGTTTGTACAAAAAAGCAGGCTTAATGGCTGAAAAATCTTCTTCCA |
| CrNPF2.4-ExtRV | GGGGACCACTTTGTACAAGAAAGCTGGGTATCAAACCACATCTAAGTT |
| CrNPF2.3-ExtFW | GGGGACAAGTTTGTACAAAAAAGCAGGCTTAATGGAGAGCAAGAATGAG |
| CrNPF2.3-ExtRV | GGGGACCACTTTGTACAAGAAAGCTGGGTATCAACCTTTAATGCCCTT |
| CrNPF2.2-ExtFW | GGGGACAAGTTTGTACAAAAAAGCAGGCTTAATGGATTCGAAGAAAACC |
| CrNPF2.2-ExtRV | GGGGACCACTTTGTACAAGAAAGCTGGGTATTAGTGTTTAATATTATC |
| CrNPF2.5-ExtFW | GGGGACAAGTTTGTACAAAAAAGCAGGCTTAATGGAGGAGAATCAAGAG |
| CrNPF2.5-ExtRV | GGGGACCACTTTGTACAAGAAAGCTGGGTATCAAACAAGAGATTTCTC |

| Supplementary Table 2 \| **Liquid chromatography setup used for 7-deoxyloganic acid, loganic acid, secologanin and loganin analysis using the Agilent 1100 Series LC coupled to a Bruker HCT-Ultra ion trap mass spectrometer**. The HPLC gradient program, mobile phases and column information are listed below. | | | |
| --- | --- | --- | --- |
| **Time (min)** | **Mobile phase B (%)** | **Gradient type** | **Flow(µL/min)** |
| **0.00-0.50** | 2 | isocratic | 200 |
| **0.50-7.50** | 2-->40 | Linear | 200 |
| **7.50-8.50** | 40-->90 | Linear | 200 |
| **8.50-11.50** | 90 | isocratic | 200 |
| **11.50-15.00** | 2 | isocratic | 300 |
| **Mobile phase A** | Water with 0,1% (v/v) HCOOH and 50µM NaCl | | |
| **Mobile phase B** | Acetonitrile with 0,1% (v/v) HCOOH | | |
| **Column** | Zorbax SB-C18 column, 5cm × 2,1mm id, 1,8μm particle size (Agilent) | | |

| Supplementary Table 3 \| **Oligonucleotide sequences used for quantitative PCR.** | | | |
| --- | --- | --- | --- |
| **Gene** | **CAROS_ID** | **Forward Primer sequence (5’ 🡪 3’)** | **Reverse Primer sequence (5’ 🡪 3’)** |
| *SAND* | Caros010066.1 | CAGTTCCACAATGCTTTCTGAC | GGGACTGATCAATCGAAGTAGC |
| *N2227* | Caros011588.1 | GGTTGCTCTTCATTACGGATTT | TGCAGCATAGTAATGGTTTTGC |
| *GES* | Caros003727.1 | GCTTTGTTTTTCACACCTTGT | CTTAGCACATTTTACTCTCTC |
| *G80* | Caros006766.1 | GTACTCTCTCCCTCATGGTTGG | CCGTCCATTACTCCCATAAAGA |
| *IO* | Caros020058.1 | CCGGTTTTCTTCTCCTCCTTAT | CCGTATTTGGACTTGAGCTTGT |
| *IS* | Caros008267.1 | TCTTGGGTTTTAGGAATTCGATGA | AAACCAAACCCAAAGCAGAAAA |
| *7DLGT* | Caros009839.1 | CCACCACAAGACCTGAAGAAAT | CTAGTCTGAGCATGGAGTTCACA |
| *7DLH* | Caros005234.1 | CCAGGCAAGGATTTCATTATTC | TTCAGTCCAAAGTCAGGCAAG |
| *LAMT* | Caros002904.1 | CACTTCTTCATCTCTCTCTTC | CAATGGAATCAATTGTGGCAAC |
| *SLS1* | Caros002866.1 | CCACTGGAGTTTTGCTCACA | TTATTCCTGCCAAAGGCTTC |
| *TDC* | Caros014930.1 | GGTTAGATCAGACTCCAGATTCG | GTCGAGTTAAGCATGTCCAAAA |
| *STR* | Caros011578.1 | TCTCTTCCATAGCTCTGTGGGTA | GCAGCAGACACTCAAAATCTCC |
| *SGD* | Caros009426.1 | TGGTCATTCTTTGACAACTTCG | TCTCTTTTTAGCCGTGTTCGTA |
| *CrNPF2.6* | Caros010208.1 | CAATATGCTTCTGAGGTACCCAAG | CAGCAATTTTGACCCCTGAGGAC |
| *CrNPF2.4* | Caros022254.1 | CATGTTATTTCTCCTACTACTCTC | GATCCTTCTGCGTATAGATGGTC |
| *CrNPF2.5* | Caros015290.1 | AAAGAATGGGAATTGGAATGG | TTCGTGGCTCAAAGCCTAAT |

| Supplementary Table 4 \| **FPKM (fragments per kilobase of exon per million fragments mapped) values of *C. roseus* genes in plant organs.** FKPM values were extracted from the ORCAE database  (http://bioinformatics.psb.ugent.be/orcae/overview/Catro) and were generated through RNA-Seq analysis on different *C. roseus* plant organs (Góngora-Castillo et al. 2012) and seedlings treated with methyl jasmonate (Van Moerkercke et al. 2013). | | | | | | | | | |
| --- | --- | --- | --- | --- | --- | --- | --- | --- | --- |
| **Caros ID** | **Gene ID** | **FL** | **ML** | **IML** | **ST** | **R** | **SLC6** | **SLMJ6** | **SLMJ24** |
| Caros010208.1 | CrNPF2.6 | 22.02 | 11.15 | 14.22 | 15.85 | 10.73 | 8.54 | 19.65 | 35.04 |
| Caros022254.1 | CrNPF2.4 | 60.49 | 7.26 | 5.93 | 2.05 | 1.80 | 10.67 | 38.99 | 32.44 |
| Caros015290.1 | CrNPF2.5 | 8.44 | 0.37 | 1.13 | 18.63 | 0.99 | 2.42 | 12.09 | 14.68 |
| Caros003727.1 | GES | 3.44 | 30.46 | 33.50 | 14.76 | 241.93 | 22.24 | 39.49 | 23.91 |
| Caros006766.1 | G8O | 10.47 | 103.09 | 123.95 | 64.34 | 498.03 | 38.60 | 67.05 | 29.98 |
| Caros003452.1 | 8HGO | 12.15 | 53.34 | 49.52 | 37.16 | 569.65 | 66.32 | 135.41 | 46.91 |
| Caros008267.1 | POR5/IS | 4.60 | 44.99 | 46.69 | 31.59 | 290.91 | 35.46 | 73.94 | 25.88 |
| Caros003676.1 | IO | 354.76 | 54.25 | 61.95 | 50.49 | 215.85 | 19.92 | 30.83 | 44.49 |
| Caros009839.1 | 7DLGT | 5.90 | 13.62 | 24.96 | 29.28 | 73.23 | 4.50 | 10.91 | 9.36 |
| Caros005234.1 | 7DLH | 29.86 | 44.85 | 62.75 | 93.59 | 134.56 | 28.23 | 92.32 | 36.67 |
| Caros002904.1 | LAMT | 26.88 | 91.33 | 139.33 | 214.13 | 57.51 | 17.77 | 192.74 | 134.91 |
| Caros002866.1 | SLS1 | 13.85 | 67.82 | 113.56 | 146.34 | 74.81 | 38.51 | 363.69 | 238.21 |
| Caros003710.1 | SLS2 | 33.78 | 66.33 | 203.73 | 475.29 | 134.20 | 69.19 | 760.95 | 485.16 |
| Caros011578.1 | STR1 | 63.98 | 76.97 | 120.54 | 190.69 | 78.28 | 104.14 | 348.46 | 268.29 |
| Caros010005.1 | STR2 | 63.28 | 26.81 | 35.52 | 17.04 | 30.69 | 32.86 | 32.45 | 17.24 |
| Caros009426.1 | SGD | 6.80 | 15.90 | 32.77 | 39.96 | 23.65 | 20.51 | 295.62 | 87.50 |
| FL, flowers; ML, mature leaf; IML, immature leaf; ST, stem; R, root; SL, seedlings, mock-treated (C) or MeJA-elicited(MJ) for 6 or 24 hours. | | | | | | | | | |

| Supplementary Table 5 \| **Oligonucleotide sequences used to generate VIGS vectors.** | | |
| --- | --- | --- |
| **Gene** | **Target** | **Primer sequence (5’ 🡪 3’)** |
| CrNPF2.4_VIGS_forward | CrNPF2.4 | GGCGCGAU TAT CTG GAT TTT GGT TAG TAC CTC AAT TGG C |
| CrNPF2.4_VIGS_reverse | CrNPF2.4 | GGTTGCGAU ATC TAA GTT CTT CTT GGG CTC ATC CAA TAT TTG |
| CrNPF2.6_VIGS_forward | CrNPF2.6 | GGCGCGAU ATG AAA CAT GTG AGA AAT TGG GAA CAA TTG G |
| CrNPF2.6_VIGS_reverse | CrNPF2.6 | GGTTGCGAU AGG CCA AGT TAC ATG GCC TAA TG |
| CrNPF2.4_VIGS_forward_fusion | CrNPF2.4 | ACTTGGACCU TAT CTG GAT TTT GGT TAG TAC CTC AAT TGG C |
| CrNPF2.4_VIGS_reverse_fusion | CrNPF2.4 | GGTTGCGAU ATC TAA GTT CTT CTT GGG CTC ATC CAA TAT TTG |
| CrNPF2.6_VIGS_forward_fusion | CrNPF2.6 | GGCGCGAU ATG AAA CAT GTG AGA AAT TGG GAA CAA TTG G |
| CrNPF2.6_VIGS_reverse_fusion | CrNPF2.6 | AGG CCA AGU TAC ATG GCC TAA TG |

| Supplementary Table 6 \| **Oligonucleotide sequences used for quantitative PCR on VIGS tissue.** | | |
| --- | --- | --- |
| **Gene** | **Target** | **Primer sequence (5’ 🡪 3’)** |
| CrNPF2.4_qPCR_forward | CrNPF2.4 | GCA ACA AAC CTC ATT AAC ATC TTC AAT GGC |
| CrNPF2.4_qPCR_reverse | CrNPF2.4 | CCC AAG GTA GAG GAA ACA GAG GCA AAG |
| CrNPF2.6_qPCR_forward | CrNPF2.6 | ACG GTA TCA CCC TAC TAC AGA GGA TGG |
| CrNPF2.6_qPCR_reverse | CrNPF2.6 | CTG ACC TTC TTG AAT TCC TAG CGT TGG |
| Rps9_qPCR_Forward | 40S ribosomal protein 9 | TTG AGC CGT ATC AGA AAT GC |
| Rps9_qPCR_Reverse | 40S ribosomal protein 9 | CCC TCA TCA AGC AGA CCA TA |
| CrNPF2.4_qPCR_forward | CrNPF2.4 | GCA ACA AAC CTC ATT AAC ATC TTC AAT GGC |
| CrNPF2.4_qPCR_reverse | CrNPF2.4 | CCC AAG GTA GAG GAA ACA GAG GCA AAG |

Supplementary figures


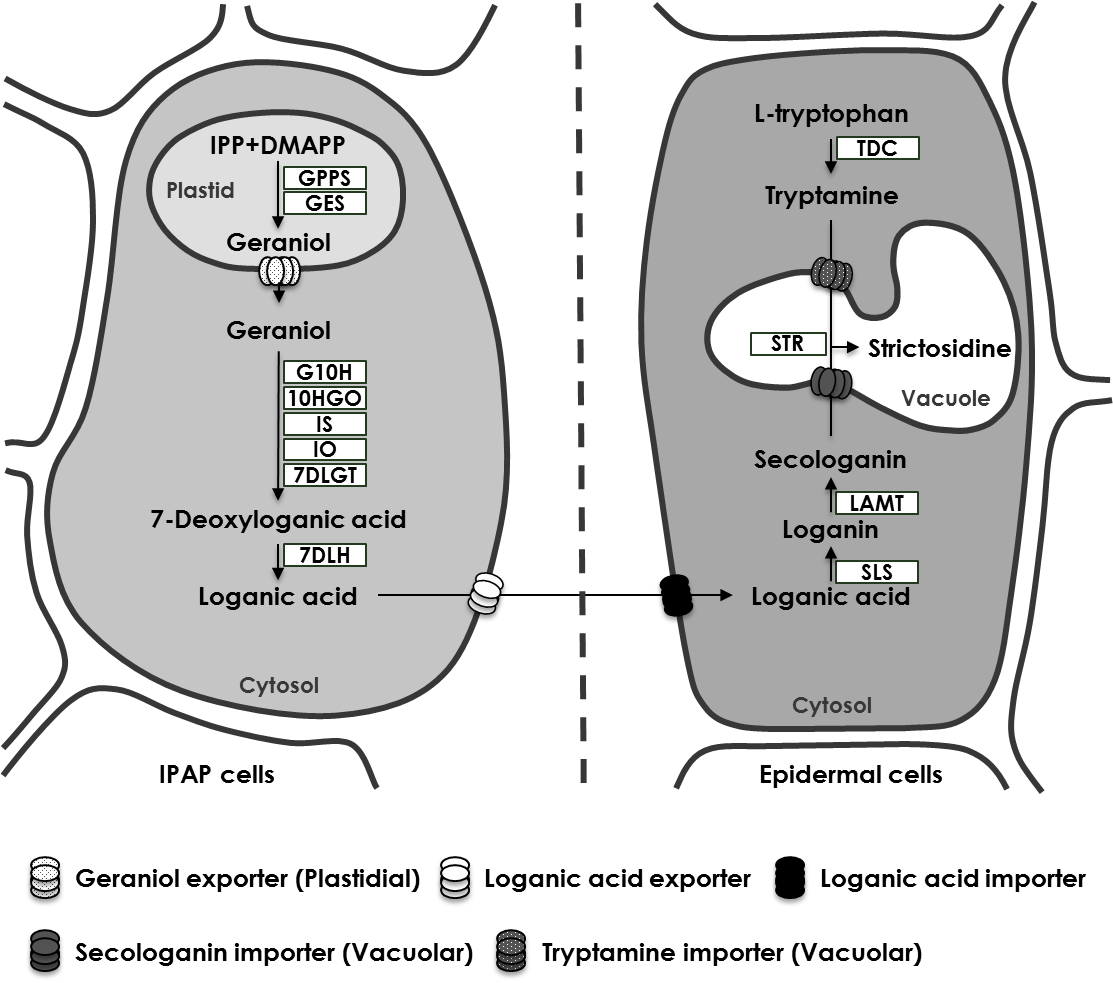


Supplementary Figure 1 | **Overview of the strictosidine biosynthetic pathway.** The model for the strictosidine pathway biosynthesis begins with geraniol formation in the plastids of IPAP cells and ends with the condensation of tryptamine and secologanin, into strictosidine, in the vacuoles of epidermal cells. Accordingly, loganic acid is the top candidate for a mobile intermediate between IPAP cells and leaf epidermal cells. Abbreviations: Isopentenyl pyrophosphate (IPP), dimethylallyl pyrophosphate (DMAPP), geranyl diphosphate synthase (GPPS), geraniol synthase (GES), geraniol 10-hdroxylase (G10H), 10-hydroxygeraniol oxidoreductase (10HGO), iridoid synthase (IS), iridoid oxidase (IO), 7-deoxyloganetic acid glucosyltransferase (7DLGT), 7-deoxyloganic acid hydrolase (7DLH), loganic acid O-methyltransferase (LAMT), secologanin synthase (SLS), L-tryptophan decarboxylase (TDC), strictosidine synthase (STR).


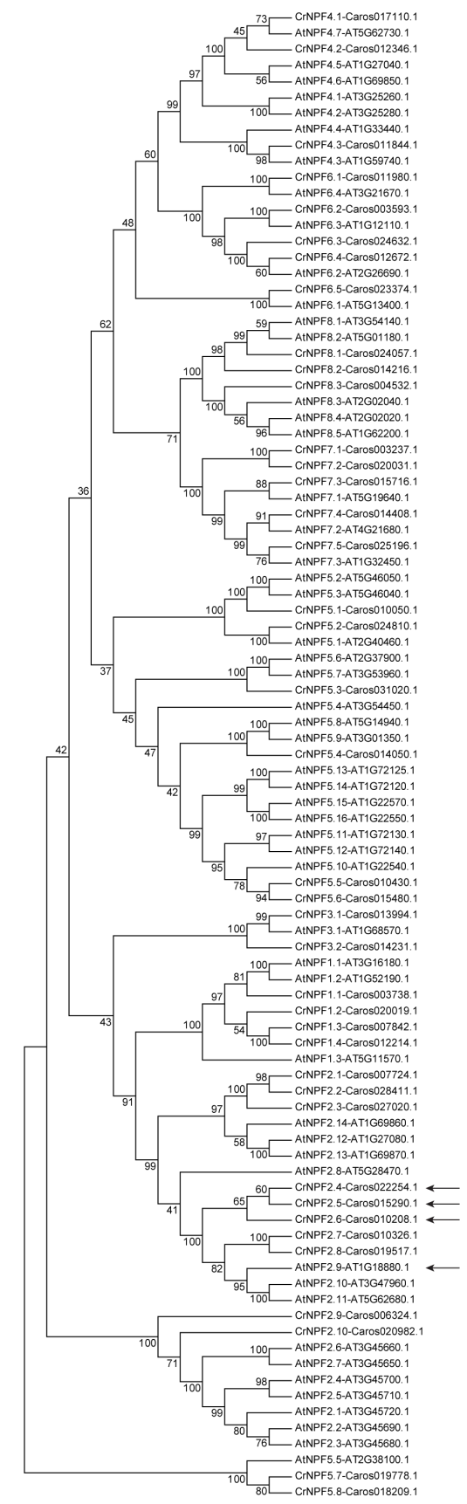


Supplementary Figure 2 | **Phylogenetic analysis of NPFs.** Phylogenetic analysis of all 53 Arabidopsis NPFs and 40 NPFs identified in the *C. roseus* transcriptome assembly (Van Moerkercke *et al.*, 2013). A bootstrap consensus tree was constructed in MEGA5 using the neighbor joining clustering method with the Jones, Taylor, and Thorton (JTT) amino acid substitution model. The bootstrap analysis was carried out with 1,000 replicates. Bootstrap confidence values are shown at the nodes. Arabidopsis and *C. roseus* NPF transporters with iridoid glucoside transport activity are indicated with arrows.

Supplementary Figure 3 | **The effects of 10-fold glucose on loganin uptake**. Loganin uptake assays were performed with and without 10-fold excess glucose in the uptake media, in order to investigate whether the transporters were glucose transporters capable of transporting iridoid glucosides (Error bars are s.e; n=4 (3X3oocytes) for all data points). The relative loganin uptake is defined as the loganin uptake into the oocytes in the presence of glucose divided by the loganin uptake in the absence of glucose. Loganin was detected by LC-MS.

Supplementary Figure 4 | **Ion dependency of loganin uptake by CrNPF2.6.** CrNPF2.6 was tested for loganin uptake in normal Kulori buffer pH5.0 (Standard), in Kulori buffer based on potassium ions (no Na^+^) and Kulori buffer based on sodium ions (no K^+^) (Error bars are s.e; n=4 (3X3oocytes) for all data points). The relative loganin uptake is defined as the loganin uptake into the oocytes divided by the loganin uptake under standard condition (Kulori buffer pH5.0). Loganin was detected by LC-MS.

Supplementary Figure 5 | **Determination of active loganin transport activity for CrNPF2.6.** CrNPF2.6 was tested for loganin uptake to determine if the transport was active or passive. CrNPF2.6 was assayed by for 20 min in media with substrate concentrations ranging from 12.5 µM to 100 µM (Error bars are s.e; n=4 (4X3oocytes) for all data points). The graph displays the loganin concentrations detected by LC-MS inside the oocytes divided by the respective media concentration. The media concentration is therefore always 1 in the graph (reference line).

**A**

**B**

Supplementary Figure 6| **Virus-induced gene silencing for *CrNPF2.4* and *CrNPF2.6*. (A)** Normalized *CrNPF2.4* and *CrNPF2.6* expression in the non-silenced VIGS-EV line, the single VIGS-CrNPF2.4 and VIGS-CrNPF2.6 silenced lines and the VIGS-CrNPF2.4 -2.6 double silenced line (Error bars are s.e., n=8 for all data points). **(B)** Untargeted metabolomics using the XCMS software package was performed for the VIGS experiment. No new peaks accumulated in the CrNPF silenced lines, relative to empty vector controls. (Error bars are s.e., VIGS-EV (n=9) VIGS-CrNPF2.4 (n=11) VIGS-CrNPF2.6 (n=8) VIGS-CrNPF2.4-2.6 double (n=11))

Supplementary Materials and Methods

**Virus-induced gene silencing of CrNPFs**

To create the vectors for virus induced gene silencing (VIGS) of the CrNPFs, the USER compatible VIGS vector pTRV2u was digested with AsiI and Nt.BbvCI. pTRV2u-CrNPF2.4 and pTRV2u-CrNPF2.6 were generated by USER cloning 200-500 bp fragments of CrNPF2.4 and CrNPF2.6, amplified from *C. roseus* cDNA using the primers in (Supplementary table 5), into the cut vector. For generation of the double VIGS vector, pTRV2u-CrNPF2.4-2.6 double, targeting both CrNPF2.4 and CrNPF2.6, USER fusion cloning was employed. *Agrobacterium* GV3101 strains containing pTRV1 (ABRC), pTRV2u-Empty Vector (pTRV2u-EV), pTRV2u-MgChl (for silencing magnesium chelatase), pTRV2u-CrNPF2.4, pTRV2u-CrNPF2.6 and pTRV2u-CrNPF2.4-2.6 double, were grown overnight in 5 ml LB supplemented with rifampicin, gentamycin and kanamycin at 28 ˚C. Cultures were pelleted at 3,000 *g*, resuspended in *Agrobacterium* inoculation solution (10 mM MES, 10 mM MgCl_2_, 200 µM acetosyringone) to an OD_600_ of 0.7, and incubated at 28 ˚C for 2 h. Strains containing pTRV2u constructs were mixed 1:1 with pTRV1 culture and this mixture was used to inoculate plants by the pinch wounding method using a pair of finely bent forceps. Plants (8-12, 2 months old) were inoculated for each construct, and the plants were grown at 25 ˚C in a 12 h photoperiod. The pTRV2u-EV plants were used as negative controls, whilst the pTRV2-MgChl plants were used as visual markers of the silencing response, with bleaching of the leaves occurring 21-25 days post inoculation. Upon silencing the plant material was harvested, ground in a Retsch ball mill under liquid nitrogen and stored at -80 ˚C before analysis by qPCR and liquid chromatography mass spectrometry (LC-MS). All of the VIGS experiment were replicated a minimum of three times per construct.

**qPCR of VIGS tissue**

For qPCR, approximately 100 mg of plant tissue was used for extraction of RNA from each replicate in a VIGS experiment using a Qiagen RNeasy plant mini kit in accordance with the manufacturer’s instructions. For each set of pTRV2u plants, a minimum of 8 plants were selected for RNA extraction and qPCR. RNA quality was assessed on a 1% agarose gel and the concentration was measured on a NanoDrop® ND-1000 (Thermo Scientific, Waltham, MA, USA). cDNA for qPCR from each replicate was synthesized using the Biorad iScript cDNA synthesis kit (BioRad, Hercules, CA, USA). For each qPCR experiment, cDNA from eight replicates of pTRV2-EV and eight replicates of pTRV2-gene of interest were used, and the qPCR reaction was performed in technical duplicates. The relative quantification of gene expression was performed using the delta delta cycle method using the 40S ribosomal protein 9 (Rps9) as a reference gene for normalisation. Each primer pair (Supplementary table 6) was used in a standard PCR reaction against *C. roseus* cDNA to ensure only one target was amplified. For each primer pair, a standard curve was generated to ensure amplification efficiency had a linear relationship with cDNA concentration, with only primer pairs giving a linear regression (*R^2^*) value of 0.99 used. The primer efficiency values generated in this study were between 99-101%.

**Liquid Chromatography Mass Spectrometry (LC-MS) of VIGS tissue**

Ground leaf tissue was weighed and collected into 200 µl methanol, containing 10 µM esculin as an internal standard, and incubated at 60 ˚C for 2 hours. After a 30 minute centrifugation step at 5000 *g,* an aliquot of the supernatant (25 µl) was mixed with an equal volume of water and analysed on a Shimadzu IT-TOF. The column used was a Phenomenex Kinetic 5 µ C18 100A (100 x 2.10 mm x 5 µm) and the binary solvent system consisted of acetonitrile (ACN) and 0.1% formic acid in water. The elution programme was as follows: 1 minute isocratic at 12% ACN, 3.5 minute gradient up to 25% ACN, 2.5 minute gradient up to 50% ACN, 1 minute gradient up to 100% ACN, 6 minute isocratic at 100% ACN, 1 minute gradient down to 12% ACN, and 2.5 minute isocratic at 12% ACN. Peak areas were calculated using LC-MS solutions and normalised by leaf mass (fresh weight) and the peak area of the internal standard.

Reference List

Góngora-Castillo, E., Childs, K. L., Fedewa, G., Hamilton, J. P., Liscombe, D. K., Magallanes-Lundback, M. et al. (2012) Development of transcriptomic resources for interrogating the biosynthesis of monoterpene indole alkaloids in medicinal plant species. *PLoS One*. Public Library of Science, 7(12), p. e52506. doi: 10.1371/journal.pone.0052506

Van Moerkercke, A., Fabris, M., Pollier, J., Baart, G. J. E., Rombauts, S., Hasnain, G. et al. (2013) CathaCyc, a metabolic pathway database built from Catharanthus roseus RNA-Seq data. *Plant and Cell Physiology*. Jpn Soc Plant Physiol, 54(5), pp. 673-85. doi: 10.1093/pcp/pct039
